# E-Cadherin Is Important in the In Vitro Postnatal Development and Function of Pig Islets

**DOI:** 10.3390/biomedicines13030627

**Published:** 2025-03-04

**Authors:** Kieran Purich, Josue Rodriguez Silva, Wenlong Huang, James Wickware, Thomas Williams, Adnan Black, Jeongbeen Kim, David Fernandez Chapa, Sudha Bhavanam, David Bigam, Daniel Schiller, Gina R. Rayat

**Affiliations:** 1 Department of Surgery, Faculty of Medicine and Dentistry, College of Health Sciences, University of Alberta, Edmonton, AB T6G 2B7, Canada; kpurich@ualberta.ca (K.P.); josuedes@yahoo.com (J.R.S.); wlhuang@stu.edu.cn (W.H.); wickware@nait.ca (J.W.); thomas7@ualberta.ca (T.W.); aablack@ualberta.ca (A.B.); jeongbee@ualberta.ca (J.K.); ddfernan@ualberta.ca (D.F.C.); bhavanam@ualberta.ca (S.B.); dbigam@ualberta.ca (D.B.); ds9@ualberta.ca (D.S.); 2 General Surgery, First Affiliated Hospital of Shantou University Medical College, Shantou 515041, China; 3 Ray Rajotte Surgical-Medical Research Institute, Faculty of Medicine and Dentistry, College of Health Sciences, University of Alberta, Edmonton, AB T6G 2S2, Canada; 4 Alberta Diabetes Institute, Faculty of Medicine and Dentistry, College of Health Sciences, University of Alberta, Edmonton, AB T6G 2E1, Canada; 5 Alberta Transplant Institute, Faculty of Medicine and Dentistry, College of Health Sciences, University of Alberta, Edmonton, AB T6G 1C9, Canada; 6 Women and Children’s Health Research Institute, Faculty of Medicine and Dentistry, College of Health Sciences, University of Alberta, Edmonton, AB T6G 1C9, Canada

**Keywords:** adult pig islets, neonatal pig islets, E-cadherin, glucose-stimulated insulin secretion, in vitro culture

## Abstract

**Background:** Pig islets have the potential to address the limited supply of human islets available for transplantation. However, the knowledge of the biology of pig islets is currently limited. Thus, this study evaluated the molecules involved in cell-to-cell adhesion and insulin secretion pathways during the in vitro development of neonatal pig islets to understand the tissue we hope to use as a possible solution to the shortage of human islets for transplantation. **Methods**: Through RT-qPCR, immunoassays, and assessments of islet function, we explored the expression of E-cadherin and its correlation with the molecules involved in the insulin secretion pathway including GTPase, RAC1, and the membrane fusion protein SNAP25 during neonatal pig islet development. **Results**: Despite no significant difference observed in gross morphology and viability, as well as variable expression of RAC1, insulin, and SNAP25 in islets from 1-, 3-, and 7-day-old neonatal pigs, there was an apparent trend towards improved function in islets obtained from 3- and 7-day-old pigs compared with 1-day-old pigs. In the presence of 30 mM KCl, the amount of insulin secreted by islets from 3- and 7-day-old pigs but not from 1-day-old pigs was increased. Disruption of E-cadherin interactions with monoclonal antibodies resulted in decreased insulin secretion capacity of islets from 3-day old pigs. **Conclusions**: Our results show that blocking E-cadherin interactions with monoclonal antibodies resulted in disrupted peri-islet capsule and impaired islet insulin secretion under high glucose conditions. Thus, E-cadherin is important in the in vitro postnatal development and function of pig islets.

## 1. Introduction

Pig islet xenotransplantation is a procedure that could help address the limited number of human organs available for islet transplantation [1]. Pig islets have been used successfully for the reversal of diabetes in animal models including non-human primates; however, few human studies have been performed [2,3,4,5]. Many previous and recent studies in the field of xenotransplantation have focused on developing methods such as the creation of genetically modified pigs to prevent immune rejection upon transplantation, with less focus placed on pig islets’ biology and development [5], which are fundamental in our understanding of the tissue we hope to use as a possible solution to the shortage of human islets for transplantation.

It is known that significant anatomic and physiological differences exist within the endocrine pancreas across species, making it important to study physiological pathways in pigs, as they likely differ from those seen in rodents and humans [6,7,8,9]. In addition, if one compares the islet cells in the neonatal pig pancreas with those seen in the adult pig pancreas, the typical structure in the adult pig islets is not observed in the neonatal pig islets. Like others, our research group believes that due to practical and biological advantages, neonatal pig islets (NPI) are the most promising source of islets for xenotransplantation [9,10,11]. Hence, a better understanding of how neonatal pig islets develop over time and the molecules involved during this process would facilitate the clinical translation of neonatal pig islet xenotransplantation.

The primary cellular pathway we are interested in initially investigating includes members within the conserved family of transmembrane adhesion molecules known as cadherins [12]. Epithelial (E) cadherins are known to play a role in endocrine pancreas development and structure in humans and rodents, but these findings have not been previously explored in pigs [13,14]. E-cadherin, encoded by the gene *CDH1*, is a transmembrane protein which plays a role in actin-linked cellular signaling pathways, and is involved in the aggregation of the islet cells [13,15,16,17]. The mechanism by which E-cadherin impacts islet function is still currently under investigation but is thought to be through its association with gap junction protein CX-36 [13,14,15,18,19,20]. To correlate E-cadherin expression with the expression of molecules involved in the insulin secretion pathway, we chose molecules of interest at different stages of the insulin secretion pathway within the beta cell. These molecules were the glucose transporter GLUT2, also known as solute carrier family 2 member 2 (SLC2A2) [21,22]; Ras-related C3 botulinum toxin substrate 1 (GTPase RAC1), which plays a key role in actin cytoskeletal remodeling during glucose-mediated insulin secretion [23,24]; and Synaptosome Associated Protein of 25 kDA (SNAP25), which allows for the fusion of insulin granules to the plasma membrane [21].

In this study, we characterized the changes seen in E-cadherin gene and protein expression during early neonatal pig islet development in vitro and accordingly correlated them with the islets’ gross morphology and expression of key molecules involved in insulin secretion, as well as with the function of islets through glucose-stimulated insulin secretion assays. We found no significant difference in the gross morphology and viability of islets from 1-, 3-, and 7-day-old neonatal pigs, as well as the variable expression of RAC1, insulin, and SNAP25 in islets obtained from these pigs. There was an apparent trend towards improved function in islets obtained from 3- and 7-day-old pigs compared with 1-day-old pigs. As such, the amount of insulin secreted by islets from 3- and 7-day-old pigs but not from 1-day-old pigs was increased under the high glucose plus 30 mM KCl condition. We also found that E-cadherin gene expression appears to be upregulated across seven days of in vitro culture. When E-cadherin mediated cell–cell adhesion is disrupted by monoclonal antibodies, the insulin secretion by islets under high-glucose conditions is impaired, indicating the importance of E-cadherin in pig islet development and function.

## 2. Materials and Methods

All research detailed in this manuscript followed the guidelines of the Canadian Council on Animal Care for all animal-related procedures and was approved by the University of Alberta’s Animal Care and Use Committee under Animal Use Protocol Number 326.

### 2.1. Neonatal Pig Islet Isolation and Culture

Neonatal Duroc/Landrace Large White F1-cross pigs of either sex were intentionally obtained at different ages (1, 3, and 7 days old) and transported to the University of Alberta’s Ray Rajotte Surgical Medical Research Institute (RRSMRI) on the day of pancreas procurement. Pancreas tissues were procured and placed in cooled Hanks Balanced Salt Solution (HBSS, H6136, Sigma-Aldrich, St. Louis, MO, USA). They were then digested using 1 mg/mL of collagenase XI (C7657, Sigma-Aldrich) for 8–15 min at 37 °C in a water bath and filtered through a 500 µm nylon screen following our standard protocols [10,25]. Following isolation, islets were placed in Ham’s F-10 Nutrient Mixture (N6635, Sigma-Aldrich) and kept under physiological conditions (i.e., 37 °C, 5% CO_2_ and 95% air). On Days 1, 3, 5, and 7, the culture media were changed, and samples of appropriate numbers of islets were taken for analysis by microscopy, viability assessment by Trypan Blue exclusion dye, RT-qPCR, and protein analyses [26]. On the 7th day of culture, islets were counted, and a glucose-stimulated insulin secretion assay was performed as described in Section 2.6.

### 2.2. Light Microscopy

Images of islets were taken on each day of culture using a Leica DMIL microscope (with a Zeiss AxioCam HRc camera, Carl Zeiss Canada Ltd., North York, ON, Canada and analyzed with AxioVision version 4.7.2., Leica Biosystems Inc., Deer Park, IL, USA).

### 2.3. Gene Expression Determined by Reverse Transcription Quantitative PCR (RT-qPCR)

Our RT-qPCR protocol was guided by the Minimum Information for Publication of Quantitative Real-Time PCR Experiments (MIQE) Guidelines [27]. At the time of sample collection, approximately 200 islet equivalents were introduced to 500 µL Trizol (15596018, Ambion Inc., Austin, TX, USA), vortexed for 30 s, and immediately placed in a −80 °C freezer. For RNA extraction, samples were thawed and processed through a series of steps involving glycogen (AM9510, ThermoFisher Scientific, Waltham, MA, USA), chloroform, isopropanol, and multiple washing steps with 70% ethanol. RNA was suspended in 40 µL of RNAse-free water supplemented with SUPERase In RNAse inhibitor (AM2696, ThermoFisher Scientific).

The sample concentration was determined using a Nanodrop spectrophotometer (NanoDrop 1000, ThermoFisher Scientific) to ensure 260/280 and 260/230 absorbance ratios of approximately 2. RNA was then treated with a Turbo-DNAse kit (AM2238, ThermoFisher Scientific). Following DNAse treatment, the sample concentration was determined again on the Nanodrop and diluted to 100 ng/µL. RNA quality was tested using two separate methods: RNA integrity (Agilent 2100 Bioanalyzer, Agilent Technologies Inc., Santa Clara, CA, USA) and by an RNAIQ Assay (Qubit spectrophotometer, ThermoFisher Scientific). RNA samples of unacceptable quality were not used.

Total RNA (600 ng) was transformed into cDNA using the Applied Biosystems High-Capacity RNA-to-cDNA kit (43874056, Applied Biosystems, Waltham, MA, USA). All RNA samples had negative RT controls completed. Samples were run on a Bio-Rad T100 thermal cycler (Life Science, Mississauga, ON, Canada) for one hour at 37 °C, followed by 5 min at 95 °C. cDNA samples were stored at −20 °C.

TaqMan primers were used for the RT-qPCR assays. Commercially available sequences were used when available, whereas for *SNAP25*, a custom TaqMan primer was designed using gene sequences obtained from the National Center for Biotechnology Information (NCBI). Gene variants were aligned using the Basic Local Alignment Search Tool (BLAST) database to ensure that all transcript variants would be detected, and the resulting primer/probe set was ordered through the custom assay design tool available from ThermoFisher. Details on the primers’ IDs and sequences are available in Tables S1 and S2.

All primers were validated in two reproducible analyses across 5-fold concentrations with efficiencies between 83.8% and 100.9%. PCR amplification assays were completed using Applied Biosystems TaqMan Fast Advanced Master Mix (4,444,557, Applied Biosystems). Each reaction had a 10 µL total volume, was manually pipetted, and run for 40 cycles on a StepOnePlus real-time PCR system (Applied Biosystems). All samples were run in triplicate; samples were manually reviewed for outliers, which were discarded (>0.5 CT value difference between triplicates); and averages were taken. Three reference genes (*Beta Actin*, *GAPDH*, and *HPRT1*), which spanned different physiological pathways were run alongside the samples. Relative gene expression was determined using multiple gene analysis, taking into consideration the primers’ efficiency, and calculated using Microsoft Excel version 16.58 [28].

### 2.4. Protein Expression Quantified by ProteinSimple’s Western Immunoassay

Protein from 2000 islet equivalents was extracted using a RIPA buffer (20-188, MilliporeSigma, Burlington, MA, USA) with 0.1% protease inhibitor (P8340, Sigma-Aldrich) following standard protocols. A Pierce BCA protein kit (23227, ThermoFisher Scientific) was used to quantify the total protein concentration. Specific protein quantification was performed using ProteinSimple’s WES machine (San Jose, CA, USA) following standard protocols. Briefly, lysate and antibody concentrations were optimized as follows: E-cadherin (lysate, 200 µg/mL; 4A2C7 antibody concentration, 1:50; Invitrogen, Waltham, MA, USA), GLUT2 (lysate, 1000 µg/mL; LS-C40343 antibody concentration, 1:50; LSBio, Seattle, DC, USA), and SNAP25 (lysate, 1000 µg/mL; ab11102 antibody concentration, 1:50; Abcam Ltd., Cambridge, UK). The intensity of binding was visualized by chemiluminescence (Figure S1). All samples were run in duplicate, and the averages of both samples were taken. Data were analyzed using ProteinSimple’s Compass for Simple West software version 6.0.0. Detected standards and peaks were fitted manually to obtain the best estimates of areas under the curve.

### 2.5. Immunostaining of Islets

Aliquots of islets from 1-, 3-, and 7-day-old neonatal pigs were collected on Days 3, 5, and 7 of culture for immunostaining. Briefly, islets were fixed in a zinc–formaldehyde solution, stored in 70% ethanol, and then embedded in agar before paraffin embedding and sectioning. Paraffin-embedded islet sections (5 µm) were stained with primary antibodies and incubated with the respective secondary antibodies (Table S3). Heat-mediated antigen retrieval was utilized for E-cadherin and SNAP25 staining using a domestic microwave. For E-cadherin staining, avidin–biotin complex/horseradish peroxidase (Vector Laboratories, Inc., Newark, CA, USA) and 3, 3-diaminobenzidinetetrahydrochloride (BioGenex, San Ramon, CA, USA) were used to detect positive-stained cells (brown color). The islet sections were counterstained with Harris’ hematoxylin. For the SNAP25 and insulin immunofluorescence staining, islets were counterstained with DAPI (D1306, Molecular Probes, Inc., Eugene, OR, USA) following the manufacturer’s protocol.

### 2.6. In Vitro Islet Function Assessment by the Glucose-Stimulated Insulin Secretion Assay

On Day 7 of culture, duplicates of 200 islet equivalents obtained from 1-, 3-, and 7-day-old neonatal pigs in Ham’s F10 medium were placed in Eppendorf tubes under physiological conditions for 1 h. Following a 1 h incubation under physiological conditions, the islet suspension was spun down at 157× *g*, and all Ham’s F10 medium supernatant was removed without disturbing the pellet. The islets were rinsed with 1.5 mL of a pre-warmed KRBH medium containing 2.8 mM glucose and placed in the incubator for 2 h. This rinsing process was repeated twice, serving as the time to allow the islets to reach a steady state in the KRBH with 2.8 mM of a glucose solution. Afterwards, Eppendorf tubes containing the islets were removed from the incubator and spun down, and the KRBH with 2.8 mM glucose solution was removed. Next, 750 µL of KRBH medium with 2.8 mM of glucose was added and incubated for 1 h, then 500 µL of the KRBH with 2.8 mM glucose solution was collected and stored at −20 °C in a freezer for an analysis of insulin secretion under low-glucose conditions. The remaining solution was discarded. After exposure to the low-glucose-containing KRBH solution, the same islets were exposed to KRBH medium containing 20 mM glucose following the method above, then the islets were exposed to KRBH medium with 20 mM glucose plus 30 mM KCl (Table S4). At the end of the incubation period, 500 µL of KRBH with glucose with or without KCl was collected and stored at −20 °C in a freezer for an analysis of insulin secretion under high-glucose condition with or without KCl. For insulin content, 750 µL of Azol buffer (obtained from a solution made from 400 mL of Milli-Q water, 57 mL of glacial acetic acid, and 1.25 g BSA) was added to the islets to liberate all the insulin from the islets. The islet suspension was vortexed for 5 s and placed in the −20 °C freezer until analysis for insulin content. The amount of insulin in each sample was analyzed using a Pig Insulin ELISA kit (10-1200-01, Mercodia, Uppsala, Sweden), and the absorbance was measured at 450 nm using a Multiskan Sky (ThermoFisher Scientific) spectrophotometer. Each sample was run in duplicate, with averages taken and shown as scatter graphs.

Additional glucose-stimulated insulin secretion assays were completed on Day 8 of culture for 3-day-old pig islets to determine the effect of blocking E-cadherin interactions on pig islet function. Islets were exposed to various experimental conditions after treatment with anti-E-cadherin monoclonal antibody (5 µg/µL). Specifically, 1000 islet equivalents were placed in Eppendorf tubes and were rinsed multiple times with KRBH medium supplemented with 2.8 mM of glucose. These islets were then placed in 500 µL of KRBH medium supplemented with 2.8 mM of glucose and incubated under physiological conditions to equilibrate for 1 h. Anti-E-cadherin monoclonal antibody was then added and incubated under physiological conditions for 90 min. The islets were then rinsed with KRBH medium supplemented with 2.8 mM of glucose multiple times and then divided into separate Eppendorf tubes each containing approximately 200 islet equivalents per condition. A similar glucose-stimulated insulin secretion assay described in the paragraph above was performed. In addition, images of islets were taken using light microscopy.

### 2.7. Statistical Analysis

Statistical analysis was completed using GraphPad Prism software (Version 8, San Diego, CA, USA). Differences between groups were identified using the Kruskal–Wallis test with Dunn’s post hoc multiple comparisons test; *p* values ≤ 0.05 between the groups compared were considered statistically significant.

## 3. Results

### 3.1. Morphology and Viability of Islets from Various Ages of Neonatal Pigs

**Figure 1:**
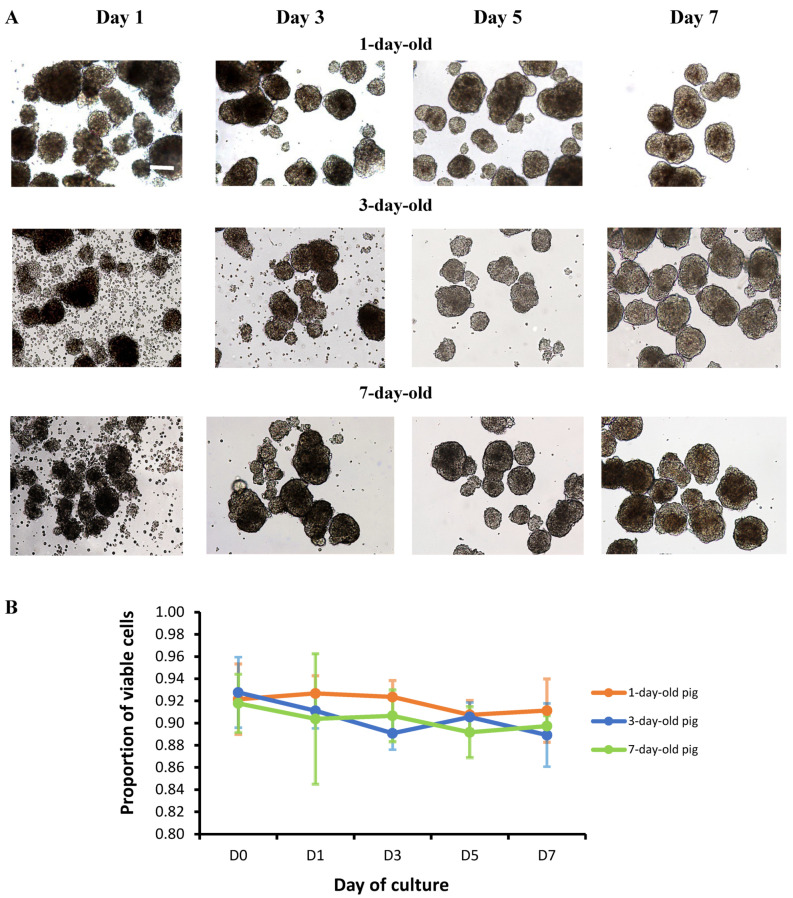
Morphology and viability of neonatal pig islets. (**A**) Light microscopic images of neonatal pig islets from 1-, 3-, and 7-day-old neonatal pigs, taken at various days of in vitro culture (Days 1, 3, 5, and 7). Images were taken at 10x objective magnification. The scale bar represents 100 µm. (**B**) Viability of islets from 1- (n = 4), 3- (n = 6), and 7-day-old pigs (n = 3) cultured for 7 days as measured by a Trypan Blue exclusion dye assay. Error bars indicate standard deviations. The *p* values are not statistically significant among the three groups compared.

On light microscopy, islets from 1-, 3-, and 7-day-old neonatal pigs were similar in gross morphology at 1, 3, 5, and 7 days of culture. The number of contaminating acinar cells detected on the day of isolation (Day 0) and Day 1 decreased to a point where they were minimal and barely visible in all cultures by Day 3 to Day 7 (Figure 1A). The formation of the peri-islet capsule, forming the “micro-organ” structure or islet cluster that has been previously discussed, was visualized starting at Day 3 [29,30,31] (Figure 1A). All islets obtained from 1-, 3-, and 7-day-old neonatal pigs had comparable viability while in culture, with no significant differences between the groups at Day 7 of culture (Figure 1B).

### 3.2. Significant Discrepancies in RNA Quality Using RIN Assessment and RNAIQ Assays

As expected, the extracted RNA from each sample in our RT-qPCR assays did not contain genomic DNA after DNAse treatment. The primers we used were found to have efficiencies between 83.8% and 100.9%. Multigene analysis was initially performed with *Beta Actin*, *GAPDH*, and *HPRT1*, and it was determined that the optimal normalization factor was achieved when using only *Beta Actin* and *GAPDH* as the reference genes. An RNA Integrity Number (RIN) assessment using the Agilent 2100 Bioanalyzer demonstrated high levels of RNA degradation at Days 0 and 1 of culture (RIN = 2.2 ± 1.0, n = 8 and 3.2 ± 2.9, n = 7, respectively, Table S5). This high level of RNA degradation was likely due to RNAses from higher amounts of contaminating acinar cells on Days 0 and 1 of culture. To ensure we were focusing on changes in RNA expression by the islets and not detecting changes due to RNA expression by the contaminating acinar cells, we decided to focus on analyzing gene expression on Days 3, 5, and 7 of culture. Interestingly, the values obtained by the RNAIQ assay did not correlate with the RIN values across all samples, with significant discrepancies occurring at low RIN values, and these discrepancies correlated with the inability to amplify and quantify RNA with RT-qPCR (Figure S2). Following this finding, RIN values were used over RNAIQ values for all subsequent samples.

### 3.3. Variable Changes in the Gene and Protein Expression of E-Cadherin During In Vitro Postnatal Development

**Figure 2:**
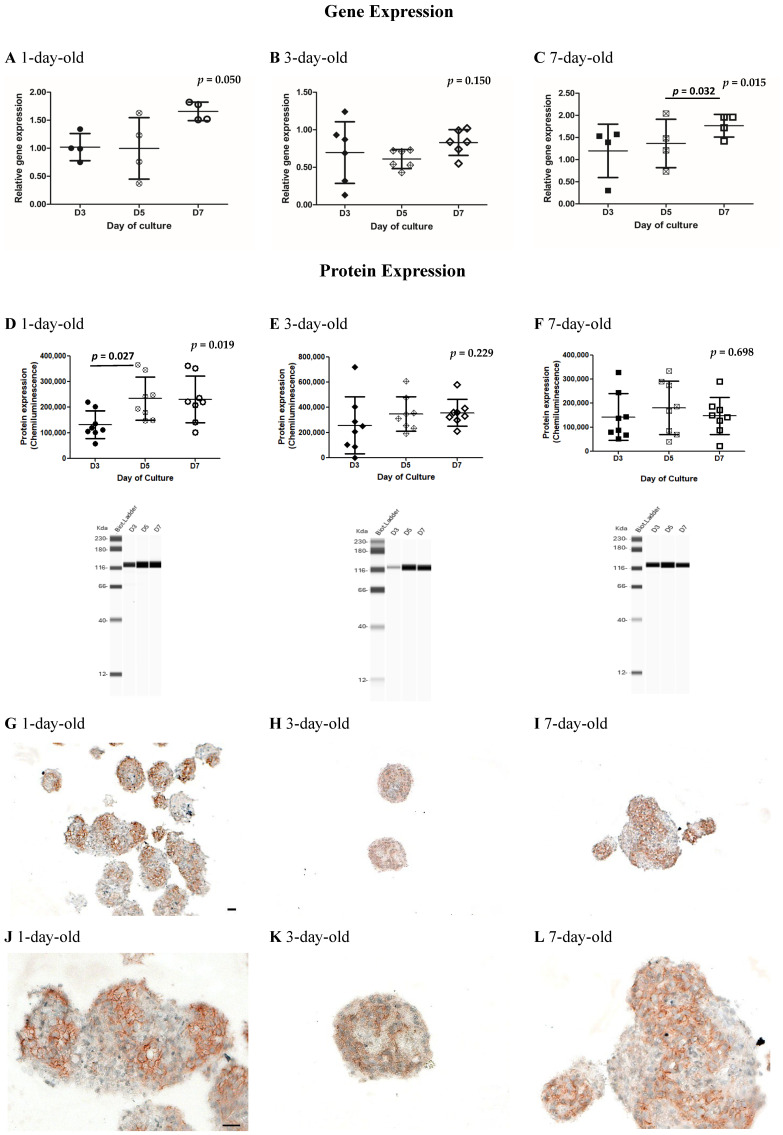
Quantification of the *CDH1* gene by RT-qPCR and E-cadherin protein expression by Western immunoassays and immunostaining in islets obtained from 1-, 3-, and 7-day-old neonatal pigs cultured for 7 days. (**A**) *CDH1* gene expression in islets from 1-day-old pigs (n = 4), (**B**) 3-day-old pigs (n = 6), and (**C**) 7-day-old pigs (n = 4); *p* = 0.050 and *p* = 0.015 between Days 3, 5, and 7 of culture in islets obtained from 1- and 7-day-old pigs, respectively, as determined by the Kruskal–Wallis test; *p* = 0.032 between Day 5 and Day 7 of culture in islets obtained from 7-day-old pigs as determined by Dunn’s multiple comparisons test. Error bars indicate standard deviations. Circles (black, white cross, and white) represent values from 1-day-old pigs on Days 3, 5, and 7 of culture, respectively (A, D). Diamonds (black, white cross, and white) represent values from 3-day-old pigs on Days 3, 5, and 7 of culture, respectively (B, E). Squares (black, white cross, and white) represent values from 7-day-old pigs on Days 3, 5, and 7 of culture, respectively (C, F). E-cadherin protein expression and representative Western immunoassay images from (**D**) 1-day-old pigs, (**E**) 3-day-old pigs, and (**F**) 7-day-old pigs (n = 8 for each group); *p* = 0.019 between Days 3, 5, and 7 of culture in islets obtained from 1-day-old pigs as determined by the Kruskal–Wallis test; *p* = 0.027 between Day 3 and Day 5 of culture in islets obtained from 1-day-old pigs as determined by Dunn’s multiple comparisons test. Error bars indicate standard deviations. Representative images of E-cadherin immunostained islets from 1-day-old pigs (**G**,**J**), 3-day-old pigs (**H**,**K**), and 7-day-old pigs (**I**,**L**). Images shown in (**G**–**I**) and (**J**–**L**) were taken at 10× and 25× objective magnification, respectively. Brown staining represents a positive stain for E-cadherin. The scale bar represents 50 µm (**G**–**I**) and 20 µm (**J**–**L**).

Statistically significant differences in *CDH1* gene expression in islets isolated from 1- (*p* = 0.050) and 7-day-old but not from 3-day-old pigs (*p* = 0.0150) were observed as determined by the Kruskal–Wallis test (Figure 2A–C, Table S6). The levels of *CDH1* gene expression were significantly increased from Day 5 to Day 7 of culture in islets obtained from 7-day-old neonatal pigs (Figure 2C, Table S6, *p* = 0.032 as determined by Dunn’s multiple comparisons test). In terms of E-cadherin protein expression (Figure 2D–F, Table S7), our results demonstrate that E-cadherin protein expression in islets obtained from 1-day-old but not from 3- and 7-day-old pigs cultured for 7 days were significantly different (*p* = 0.019 as determined by the Kruskal–Wallis test). Further analysis revealed that E-cadherin protein expression from 1-day-old pigs cultured for 3 and 5 days was statistically significantly different (*p* = 0.027 as determined by Dunn’s multiple comparisons test, Figure 2D, Table S7). Immunostaining revealed the presence of E-cadherin on the cell membrane of clusters of islet cells (Figure 2G–L). E-cadherin was localized on the plasma membrane, with strong expression between the junction of these cells (Figure 2G–L).

### 3.4. Variable Changes in the Gene Expression of RAC1 and Insulin During In Vitro Postnatal Development

**Figure 3:**
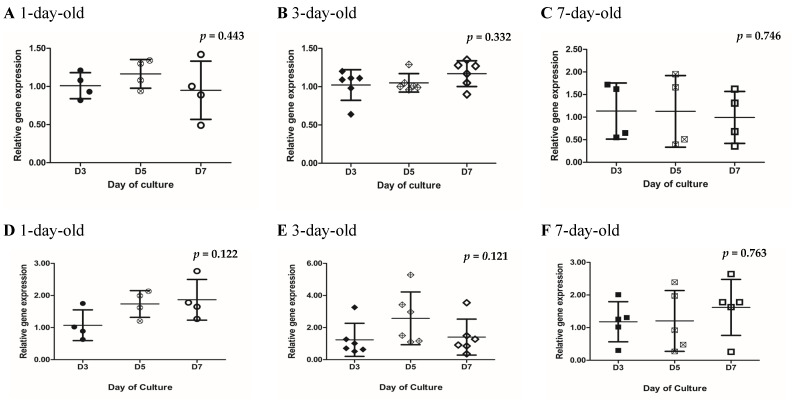
Quantification of *RAC1* and *insulin* gene expression in islets obtained from 1-, 3-, and 7-day-old neonatal pigs cultured for 7 days. *RAC1* gene expression in islets from 1-day-old pigs (**A**) (n = 4), 3-day-old pigs (**B**) (n = 6), and 7-day-old pigs (**C**) (n = 5). *Insulin* gene expression in islets from 1-day-old pigs (**D**) (n = 4), 3-day-old pigs (**E**) (n = 6), and 7-day-old pigs (**F**) (n = 5). Error bars indicate standard deviations; *p* = 0.443 for 1-day-old pigs, 0.332 for 3-day-old pigs, and 0.746 for 7-day-old pigs between *RAC1* groups, and *p* = 0.122, 0.121, and 0.763 for 1-, 3-, and 7-day-old pigs, respectively, for *Insulin* groups, as determined by the Kruskal–Wallis test. Circles (black, white cross, and white) represent values from 1-day-old pigs on Days 3, 5, and 7 of culture, respectively (A, D). Diamonds (black, white cross, and white) represent values from 3-day-old pigs on Days 3, 5, and 7 of culture, respectively (**B**,**E**). Squares (black, white cross, and white) represent values from 7-day-old pigs on Days 3, 5, and 7 of culture, respectively (**C**,**F**).

*RAC1* gene expression levels in islets obtained from 1-, 3-, and 7-day-old neonatal pigs were comparable across Day 3, Day 5, and Day 7 of culture with no statistically significant differences being detected among the groups (Figure 3A–C, Table S8). The *insulin* gene expression levels in islets obtained from different ages of neonatal pigs showed variable fluctuations and were not statistically different among the groups compared (Figure 3D–E, Table S9).

### 3.5. Variable Changes in the Gene and Protein Expression of SNAP25 During In Vitro Postnatal Development

**Figure 4:**
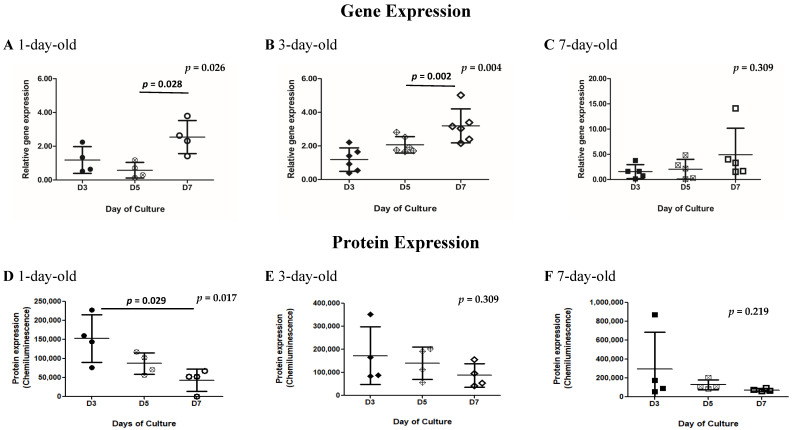

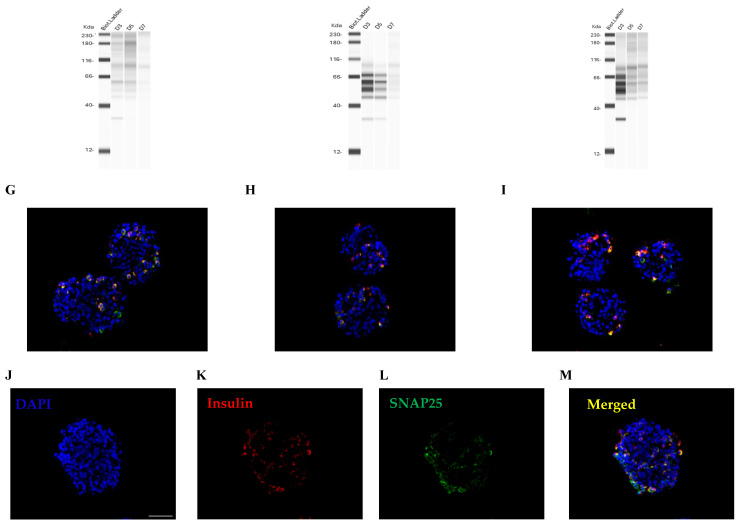
Quantification of *SNAP25* gene and SNAP25 protein expression in islets obtained from 1-, 3-, and 7-day-old neonatal pigs cultured for 7 days. *SNAP25* gene expression in islets from 1-day-old pigs (**A**, n = 4), 3-day-old pigs (**B**, n = 6), and 7-day-old pigs (**C**, n = 4); *p* = 0.026 and *p* = 0.004 between islets from 1- and 3-day-old neonatal pigs cultured for 3, 5, and 7 days as determined by the Kruskal–Wallis test; *p* = 0.028 between islets obtained from 1-day-old pigs cultured for 5 and 7 days as determined by Dunn’s multiple comparisons test; *p* = 0.002 between islets obtained from 3-day-old pigs cultured for 5 and 7 days as determined by Dunn’s multiple comparisons test. Error bars indicate standard deviations. SNAP25 protein expression and representative Western immunoassay images in islets from 1-day-old pigs (**D**, n = 4), 3-day-old pigs (**E**, n = 4), and 7-day-old pigs (**F**, n = 4); *p* = 0.017 between islets from 1-day-old pigs as determined by the Kruskal–Wallis test; *p* = 0.029 between islets from 1-day-old pigs cultured for 3 and 7 days. Error bars indicate standard deviations. Circles (black, white cross, and white) represent values from 1-day-old pigs on Days 3, 5, and 7 of culture respectively (**A**,**D**). Diamonds (black, white cross, and white) represent values from 3-day-old pigs on Days 3, 5, and 7 of culture respectively (**B**,**E**). Squares (black, white cross, and white) represent values from 7-day-old pigs on Days 3, 5, and 7 of culture respectively (**C**,**F**). Representative merged images of SNAP25 and insulin immunostained islets from 1 day-old (**G**), 3-day-old (**H**), and 7-day-old pigs (**I**). (**J**–**M**) Representative immunostained images of islets from 7-day-old pigs on Day 7 of culture showing DAPI in blue (**J**), insulin in red (**K**), SNAP25 in green (**L**), and the merged image with all three colors (**M**). Scale bar represents 50 µm.

*SNAP25* gene expression in islets from 1-, 3-, and 7-day-old neonatal pigs appeared to increase from Day 3 to Day 7 of culture (Figure 4B,C, Table S10). This increasing trend was found to be statistically significant in islets from 1- and 3-day-old pigs (*p* = 0.026 and *p* = 0.004, respectively, as determined by the Kruskal–Wallis test; Figure 4A,B, Table S10). A similar trend was observed in islets from 7-day-old pigs, but this was not found to be statistically significant (Figure 4C, Table S10). Further analysis revealed statistically significant differences in the expression of *SNAP25* gene in islets from 1-day-old pigs cultured for 5 and 7 days (*p* = 0.028 as determined by Dunn’s multiple comparisons test, Figure 4A, Table S10) and islets from 3-day-old pigs cultured for 5 and 7 days (*p* = 0.002 as determined by Dunn’s multiple comparisons test, Figure 4B, Table S10). For SNAP25 protein expression, a decreasing trend was apparent across all ages of pigs (Figure 4D–F, Table S11). This decreasing trend in SNAP25 protein expression reached significance in islets from 1-day-old pigs (*p* = 0.017 as determined by the Kruskal–Wallis test), particularly between islets cultured for 1 and 7 days (*p* = 0.029 as determined by Dunn’s multiple comparisons test). Immunostaining of islets revealed co-localization of SNAP25 and insulin in most of the beta cells (Figure 4G–M).

We were also interested in determining the gene expression of glucose transporters in neonatal pig islets. We initially determined the gene expression of glucose transporter 2 (*GLUT2*), since this is the most common and widely investigated transporter of glucose in rodent and human islets. We found that *GLUT2* gene expression in neonatal pig islets was low, leading to prohibitively high CT values on RT-qPCR. As such, we were unable to confidently quantify differences among samples and have not reported them in this manuscript.

### 3.6. In Vitro Insulin Secretory Capacity of Islets Obtained from 1-, 3-, and 7-Day-Old Neonatal Pigs

**Figure 5:**
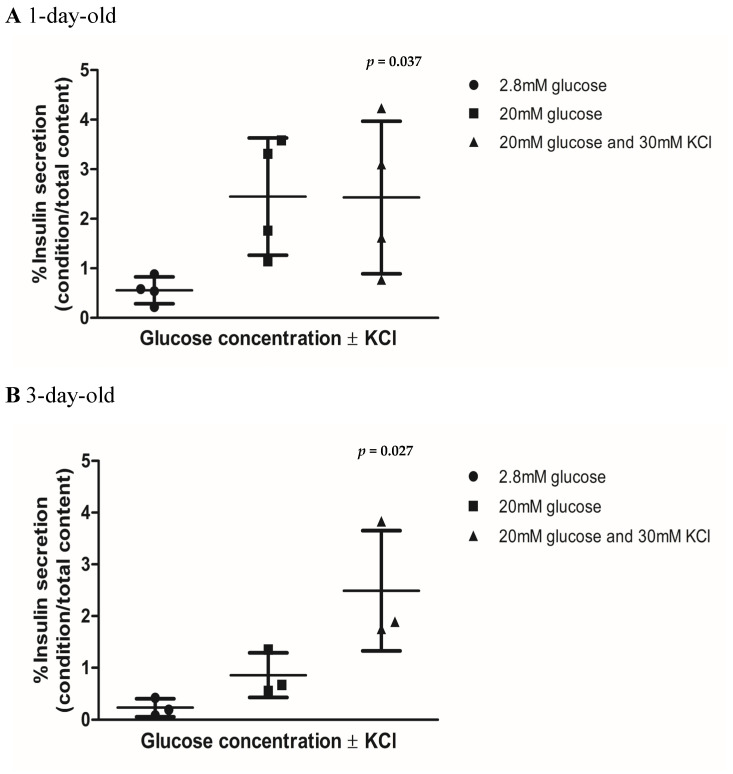

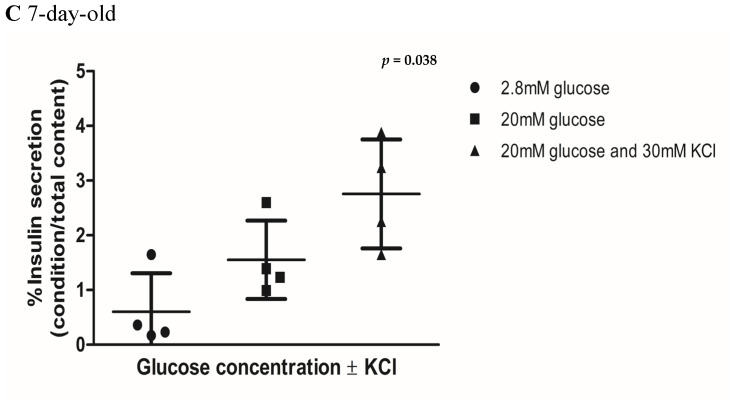
In vitro insulin secretory capacities of islets from 1-, 3-, and 7-day-old neonatal pigs at Day 7 of culture. (**A**) Islets from 1-day-old pigs (n = 4, *p* = 0.037, black circles), (**B**) 3-day-old pigs (n = 3, *p* = 0.027, black squares), and (**C**) 7-day-old pigs (n = 4, *p* = 0.038, black triangles). In total, 200 islet equivalents for each group were exposed to 2.8 mM of glucose to quantify basal insulin secretion and later exposed to 20 mM of glucose and 20 mM of glucose plus 30 mM of KCl conditions to quantify stimulated insulin secretion. Error bars indicate standard deviations. The *p* values were determined by the Kruskal–Wallis’s test.

The insulin secretory capacities of islets from 1-, 3-, and 7-day-old pigs cultured for 7 days are shown in Figure 5. Islets from 1-day-old pigs showed a significantly greater proportion of insulin secreted when stimulated with 20 mM glucose and 20 mM glucose plus 30 mM KCl compared with islets exposed to 2.8 mM glucose (*p* = 0.037 as determined by the Kruskal–Wallis test, Figure 5A, Table S12). However, the amount of insulin detected when islets from 1-day-old pigs were stimulated with 20 mM glucose plus KCl remained similar to that observed when islets were stimulated with 20 mM glucose without KCl (Figure 5A, Table S12). In contrast, islets from 3-day-old and 7-day-old pigs demonstrated progressively higher proportions of insulin secreted when stimulated with 2.8 mM glucose, 20 mM glucose and 20 mM glucose plus KCl (*p* = 0.027 and *p* = 0.038, respectively, as determined by the Kruskal–Wallis test, Figure 5B,C, Table S12). This suggests that islets from 1-day-old pigs may be deficient in regulating the response to glucose challenge compared with islets from older neonatal pigs.

### 3.7. Treatment of Islets with Anti E-Cadherin Monoclonal Antibody Resulted in Reduced In Vitro Insulin Secretory Capacity

**Figure 6:**
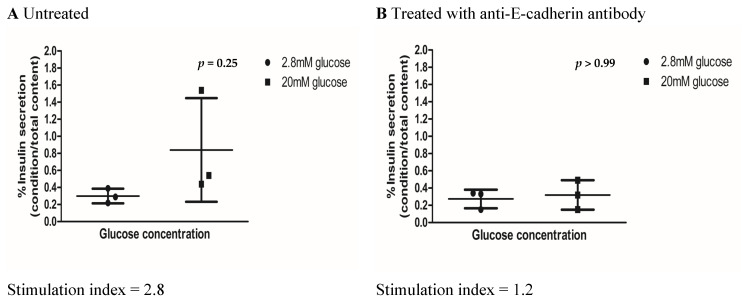

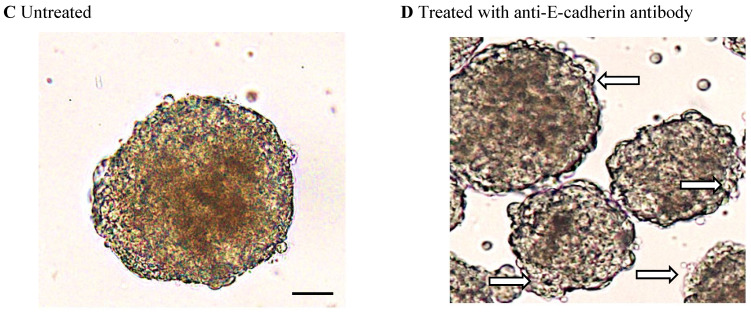
Quantitative response and images of islets from 3-day-old neonatal pigs at 8 days of culture after treatment with anti E-cadherin monoclonal antibody. (**A**) In vitro insulin secretory capacities of untreated islets (n = 3, *p* = 0.250) and (**B**) islets treated with 5 µg/µL of anti-E-cadherin monoclonal antibody (n = 3, *p* = 0.462). The *p* values were calculated by Wilcoxon’s matched pairs signed rank test. Stimulation index is calculated by the percentage of insulin secretion under the 20 mM glucose condition (black squares) divided by insulin secretion under the 2.8 mM glucose condition (black circles). Image of untreated islets (**C**) and islets treated with 5 µg/µL of anti-E-cadherin monoclonal antibody (**D**). White arrows show the disrupted peri-islet capsule in islets treated with the anti-E-cadherin monoclonal antibody. The scale bar represents 20 µm.

To demonstrate the importance of E-cadherin and cell–cell adhesion in maintaining the function of neonatal pig islets, we treated islets from 3-day-old pigs with anti-E-cadherin monoclonal antibody, subsequently testing their insulin secretory capacity in vitro. Following treatment with the anti-E-cadherin antibody, islets were not able to increase the proportion of insulin that they secreted under high-glucose conditions (Figure 6B, Table S13), demonstrating a stimulation index of 1.2-fold compared with the 2.8-fold increase seen in the untreated control group (Figure 6A,B, Table S13). In addition, following treatment with the anti-E-cadherin monoclonal antibody, islets showed qualitative changes, having a less well-defined peri-islet capsule (Figure 6D) compared with untreated islets (Figure 6C).

## 4. Discussion

Our work explored the qualitative and quantitative aspects of early neonatal pig islet biology, with special interest in cell–cell adhesion and the insulin secretion pathway, ultimately demonstrating the importance of E-cadherin in the insulin secretion pathway. In terms of the qualitative aspect of our study, we found that islets from 1-, 3-, and 7-day-old pigs displayed a similar gross morphology while in culture for 7 days. Despite differences in the age of the neonatal pigs, the islet cells aggregated to form islet clusters with peri-islet capsule (Figure 1A). This ability to reorganize in vitro has also been demonstrated for both neonatal [32] and adult [33,34] rat islet cells and is currently being explored in stem cell-derived beta cell clusters for the treatment of Type 1 diabetes. We also demonstrated the excellent viability of our islets in culture (Figure 1B), around 90%, which is comparable with that previously cited for neonatal pig islets, supporting the quality of our culture methods [11].

Few previous studies have explored genetic expression in the early neonatal phase of pig islet development; hence we also presented the quantitative aspects of neonatal pig islets’ biology by measuring the gene and protein expression of the molecules of interest. We feel that changes in gene expression experienced during the early pig islet developmental period are important to identify, as in many xenotransplantation models, neonatal pig islets are transplanted following an arbitrary number of days in culture. When working with pancreatic tissue, which is a common source of RNAses, we encountered difficulties with RNA degradation, specifically at Days 0 and 1 of culture when there were still significant numbers of acinar cells visible, as is appreciated in Figure 1A [35,36,37]. Following our experience, we highly recommend routine RNA integrity assessments when working with samples extracted from pig pancreatic tissue. Our study also highlights discrepancies we encountered between RIN and RNAIQ, which are touted as two credible assays for RNA assessment [36,38,39]. In our hands, we found that the RNAIQ assay did not always correlate with the RIN values, which is commonly treated as the gold standard for RNA degradation assessments [36]. Differences in the quality assessments occurred primarily at times of high RNA degradation, resulting in higher than expected CT values on RT-qPCR, which could be a confounding factor in RT-qPCR results if solely the RNAIQ assay was used. We encourage all groups interested in using the RNAIQ assay and Qubit spectrophotometer to assess RNA quality to ensure their assay is validated and compared across a variety of RNA levels of degradation before use.

Recently, a study by Kim et al. explored broad gene expression trends with RNA-Seq libraries obtained from pig islets across fetal and select neonatal time periods [9]. We feel our study complements their data, with our manuscript looking at additional timepoints in the early neonatal phase to identify trends in the expression of our molecules of interest over the first few days in culture. A further advanced understanding of the changes in gene and protein expression occurring in these pig islets may allow us to better predict the optimal time at which to transplant pig islets from culture into recipients. In terms of *CDH1* gene expression, we found the trend towards an increase from Day 5 to Day 7 of culture (Figure 2A–C), while the increase in E-cadherin protein expression appeared to stabilize from Day 5 to Day 7 of culture. This may be related to the maturation of the islet cell clusters inside a peri-islet capsule. A highly regulated amount of E-cadherins may be required in maintaining the balance between islet cell proliferation and the cell-to-cell adhesion properties of E-cadherins. Wakae-Takada et al. found a two-fold increase in E-cadherin in beta cells in a mouse model between birth and 22 days after birth, at which time gene expression became constant, demonstrating a somewhat similar trend to our results [18]. It would be interesting to see whether E-cadherin expression in our in vitro model would also reach a plateau beyond 7 days of culture (i.e., an additional 8 days of culture for a total of 22 days from birth). When considering the time that islets stay in the natural pancreas environment for 7 days, one would expect that the maturation of islets during this time would be more advanced than those islets obtained from pigs one day after birth.

In terms of *RAC1*, we found no significant differences in the gene expression among islets cultured for 3, 5, and 7 days (Figure 3A–C). The gene expression in islets from 3-, 5-, and 7-day-old pigs was comparable throughout the in vitro culture period. RAC1 may be one of the molecules that is critical to remain constant during islet development, since RAC1 is involved in cell motility by reorganizing the cytoskeleton in response to various stimuli and it also plays a crucial role in cell proliferation, differentiation, and migration. It would be interesting to see if the same trend is observed in RAC1 protein expression during neonatal pig islet development. We attempted to examine RAC1 protein expression by immunohistochemistry staining through enzyme–substrate reactions. However, the antibody we used did not consistently provide reliable protein expression due to the non-specific staining observed in islet samples.

Our results in terms of *insulin* gene expression in islets from 1- and 3-day-old pigs were unexpected. We anticipated that as islets developed into mature islet clusters, their insulin expression would increase. We observed this trend in islets from 7-day-old pigs but not in islets from younger pigs (Figure 3D–F). However, we found this trend to be not significantly different. It is interesting to note that the insulin gene expression in islets from 1-day-old pigs remained the same from Day 5 to Day 7 of culture (Figure 3D). We also observed the same trend in the insulin secretory capacity of these islets when stimulated with high glucose with or without KCl (Figure 5A). It appears that islets from 1-day-old pigs have a threshold in terms of insulin production upon stimulation with high glucose. While a variable trend was observed in islets from 3-day-old pigs, with an increasing trend in gene expression seen on Day 5 of culture (Figure 3D). This result is puzzling and warrants further investigation.

*GLUT2* was not highly expressed in neonatal pig islets, which is supported by previous studies [22]. As mentioned previously, we were also interested in determining *GLUT2* gene expression, since this is the most common and widely investigated transporter of glucose in rodent and human islets. In the future, it would be interesting to determine which isotype of glucose transporters are highly expressed in neonatal pig islets. The *SNAP25* gene expression in neonatal pig islets appeared to increase from Day 3 to Day 7 of culture (Figure 4A–C), which makes sense, given the increase in secretory capacities observed in islets from older pigs [11,18]; however, in our study, the protein expression did not follow a similar pattern (Figure 4D–F). SNAP25 is a protein commonly involved in synaptic vesicles targeting fusion and exocytosis and undergoes a variety of protein–protein interactions forming numerous multimeric protein complexes, which have potential to confound Western immunoassay results [40,41]. This was demonstrated in our results (Table S11 continued), as at later timepoints in culture (from Day 3 to Day 7), our Western immunoassay demonstrated a variety of bands, likely due to post-translational modifications [40,41]. Without performing immunoprecipitation and separating the free SNAP25 epitope from the attached proteins, it is difficult to properly quantify protein expression for this molecule.

In terms of neonatal pig islet function, we observed that while the islets behave in similar way in terms of forming the islet clusters (Figure 1A), their ability to respond to glucose stimulation in vitro differs (Figure 5A–C). Islets from 1-day-old pigs do not seem to follow the expected secretion pattern in vitro compared with islets from 3- and 7-day-old pigs, particularly under the high-glucose plus KCl condition (Figure 5A–C, Table S12). This may be partially due to the differences in the sensitivity of certain molecules involved in the insulin response to glucose stimulation. For example, reductions in the voltage-gated potassium (Kv) channel current are associated with increases in glucose-stimulated electrical activity and insulin secretion [42]. We recently found that islets from 7-day-old neonatal pigs displayed reduced Kv channel activity comparable with adult pig islets, which may contribute to their improved response in lowering glucose when challenged [43]. In addition, the amount of insulin secreted by islets from 1-day-old pigs tend to be higher under high-glucose conditions compared with those observed in islets from 3- and 7-day-old pigs (Figure 5A–C, Table S12). Kim et al. also showed that the basal and stimulated rate of insulin secretion decreases with time when comparing late fetal pig islets with islets obtained from 22-day-old pigs [9]. We observed the same pattern in stimulated but not in the basal rate of insulin secretion. We found that islets from 3-day-old pigs secreted less insulin compared with islets from 1-day-old pigs under low-glucose conditions (Figure 5A–C, Table S12). These findings, taken together, suggest the possibility for dysregulated insulin secretion under high-glucose conditions in islets obtained from 1-day-old pigs compared with older pigs.

Finally, previous studies evaluating the impact of E-cadherin on insulin secretion in cell lines suggest that E-cadherin alterations negatively impacted stimulated insulin secretion, likely through its effect on the actin cytoskeleton [19]. To link E-cadherin and neonatal pig islet function, we inhibited the interactions of E-cadherins using monoclonal antibodies, subsequently assessing the function and morphology of neonatal pig islets (Figure 6A–D). Like the results seen previously in MIN6 cells [31,44,45], we observed that the pig islet clusters become more loosely aggregated (Figure 6D) and have a decreased ability to increase insulin secretion under high-glucose conditions (Figure 6B). We are the first to demonstrate these findings in pig islets, which is more applicable to the field of xenotransplantation than previous studies, as it is known that clonal cell lines forming pseudo-islet structures in vitro do not always follow similar physiologic pathways as native islets [46]. This physiologic alteration and decreased ability for islets inhibited by E-cadherin monoclonal antibodies to respond to high-glucose conditions may be related to the loss of gap junction communication, as was suggested in MIN6 beta cells, but requires further study [44].

Rouiller et al., in 1991, also found that uvomorulin (now more commonly known as E-cadherin), when inhibited by monoclonal antibodies, led to the inability of rat islets to aggregate in culture, suggesting that E-cadherin was a major determinant of cell–cell adhesion within the islet, with cell–cell adhesion and communication being known to be critical for islet function [16,47,48]. In 1996, Dahl et al. explored the role of E-cadherin on islet organization in vivo using a transgenic prenatal mouse model, demonstrating the inability for pancreatic endocrine cells to cluster when E-cadherin protein was truncated [13]. In our investigation, we found that treatment of neonatal pig islets with anti-E-cadherin monoclonal antibody resulted in disruption of the peri-islet capsule (Figure 6D). It would be of interest to utilize electron microscopy to further define the structural alterations and the extent of the antibody-induced disruption of the peri-islet capsule. We speculate that this, in turn, affects cell-to-cell communication in the islet cell clusters, resulting in impaired insulin secretion upon stimulation with a high glucose concentration. Beyond promoting cell–cell adhesion and cell clustering, E-cadherin also appears to play a role in pancreatic beta cell proliferation, and overexpression of E-cadherin has been shown to limit the proliferation of pancreatic beta cell lines [15,18,29]. Further research in rodent models demonstrated multiple types of cell adhesion molecules directing differential cell segregation within islets; however, studies have not been previously completed in pigs [16,30,31]. In the future, it would be interesting to explore the role of other cell adhesion molecules during neonatal pig islet development and which specific pathway in insulin secretion might be affected when their interactions are disrupted.

During our investigation, we encountered various limitations. The range of molecules we were able to test was limited by the unavailability of pig-specific monoclonal antibodies and RNA primers for some of our molecules of interest. In addition, even some of the molecules of interest which we were able to investigate have limitations, for example, we were not able to evaluate the RAC1 protein expression due to the non-specific staining we observed with antibody to RAC1. In the future, we plan to investigate the gene and protein expression of additional molecules such as beta catenin, ephrinA5, and VAMP2, as they are known to be associated with cadherins and the actin cytoskeleton [13,21,29,40,41,49,50]. In addition, in retrospect, it would have been interesting to initially perform a broader scope of gene expression analysis through next-generation sequencing techniques like RNA-Seq to guide the selection of our molecules of interest. We also encountered difficulties with the downregulation of cadherin molecules through siRNA transfection and were unable to obtain a siRNA sequence that could downregulate pig cadherin mRNA. We eventually overcame this by using monoclonal antibodies targeting E-cadherin to inhibit its function. Lastly, we did not separate our culture preparation by islet cell type. This was preferred, as our islet products were felt to be more representative of the islet preparations which are transplanted into animal models, but we acknowledge that heterogeneity could exist between culture samples with varying levels of endocrine cells throughout different days of culture.

## 5. Conclusions

Despite similarities in the gross morphology of islets from 1-, 3-, and 7-day-old neonatal pigs, we found novel findings in the gene expression patterns across specific molecules of interest in early neonatal pig islet development. Specifically, we found that E-cadherin gene expression appears to be upregulated across seven days of in vitro culture and that E-cadherin-mediated cell–cell adhesion appeared to be important for neonatal pig islets’ insulin secretion under high-glucose conditions. Further understanding of pig islet biology is important for the advancement of pig islet xenotransplantation to the clinical setting.

## Data Availability

The raw data supporting the conclusions of this article will be made available by the authors on request.

## Supplementary Materials

The following supporting information can be downloaded at https://www.mdpi.com/article/10.3390/biomedicines13030627/s1. Figure S1: Visual demonstration of the output and interpretation of the simple Western WES machine results, interpreted using Compass Software Version 5.0.1. Figure S2: Comparison of two independent assessments used to determine RNA quality across 15 samples. Table S1: TaqMan real-time polymerase chain reaction primer details ordered off the shelf from ThermoFisher Scientific. Table S2: TaqMan real-time polymerase chain reaction primer details custom-designed by our research team. Table S3. Reagents used for immunostaining of paraffin-embedded islet sections. Table S4. KRBH solution recipe. Table S5. Average RNA Integrity Number (RIN) values of RNA extracted from islets across various days of culture. Table S6. *CDH1* gene expression ratios in islets obtained from 1-, 3- and 7-day-old pigs. Table S7. CDH1 protein expression (chemiluminescence values) in islets obtained from 1-, 3- and 7-day-old pigs. Table S7 continued. Simulated western blot gels created from Protein Simple Wes Machine demonstrating E-cadherin protein expression (chemiluminescence values) outlined in Table S7 in islets obtained from 1-, 3- and 7-day-old pigs. Table S8. *RAC1* gene expression ratios in islets obtained from 1-, 3- and 7-day-old pigs. Table S9. *Insulin* gene expression ratios in islets obtained from 1-, 3-, and 7-day-old pigs. Table S10. *SNAP25* gene expression ratios in islets obtained from 1-, 3-, and 7-day-old pigs. Table S11. SNAP25 protein expression (chemiluminescence values) in islets obtained from 1-, 3-, and 7-day-old pigs at various time points in culture. Table S11 continued. Simulated western blot gels created from Protein Simple Wes Machine demonstrating SNAP25 protein expression (chemiluminescence values) outlined in Table S11 in islets obtained from 1-, 3-, and 7-day-old pigs. Table S12. Amount of insulin secreted (over total insulin content) expressed as percent insulin secreted by islets obtained from 1-, 3-, and 7-day-old-pigs following in vitro glucose stimulation assay. Table S13. Amount of insulin secreted (over total insulin content) expressed as percent insulin secreted by untreated or E-cadherin antibody-treated islets obtained from 3-day-old pigs following in vitro glucose stimulation assay.
