# Supplementary material for: E-Cadherin Is Important in the In Vitro Postnatal Development and Function of Pig Islets
Source: Biomedicines. 2025 Mar 4;13(3):627. doi: 10.3390/biomedicines13030627 (PMC11940345; doi:10.3390/biomedicines13030627)
Supplement: Supplementary file 1 [file biomedicines-13-00627-s001.zip › biomedicines-3384214-supplementary.pdf]

# *Supplementary Materials*

**Table S1.** TaqMan real-time polymerase chain reaction primer details ordered off the shelf from ThermoFisher Scientific

| Primer Target Molecule | ThermoFisher Scientific Primer ID | Efficiency (%) | Amplicon Length (number of base pairs) |
|---|---|---|---|
| E-cadherin | Ss03377287_u1 | 88.7 | 121 |
| Insulin | Ss03386682_u1 | 100.0 | 96 |
| GLUT2 | Ss3385240_u1 | 95.7 | 79 |
| RAC1 | Ss06942696_m1 | 100.9 | 65 |
| Beta actin | Ss03376563_uH | 95.7 | 79 |
| GAPDH | Ss03375629_u1 | 92.0 | 64 |
| HPRT1 | Ss03388273_m1 | 92.9 | 112 |

**Table S2.** TaqMan real-time polymerase chain reaction primer details custom-designed by our research team

| Primer Target Molecule | Custom Primer/Probe Sequence | Efficiency (%) | Amplicon Length (number of base pairs) |
|---|---|---|---|
| SNAP25 | Forward primer<br>AATCAGGATGGAGTT-GTGGCCA<br>Reverse primer<br>CATGGAC-GGAGGTTTCCAATGATG | 83.8 | 168 |

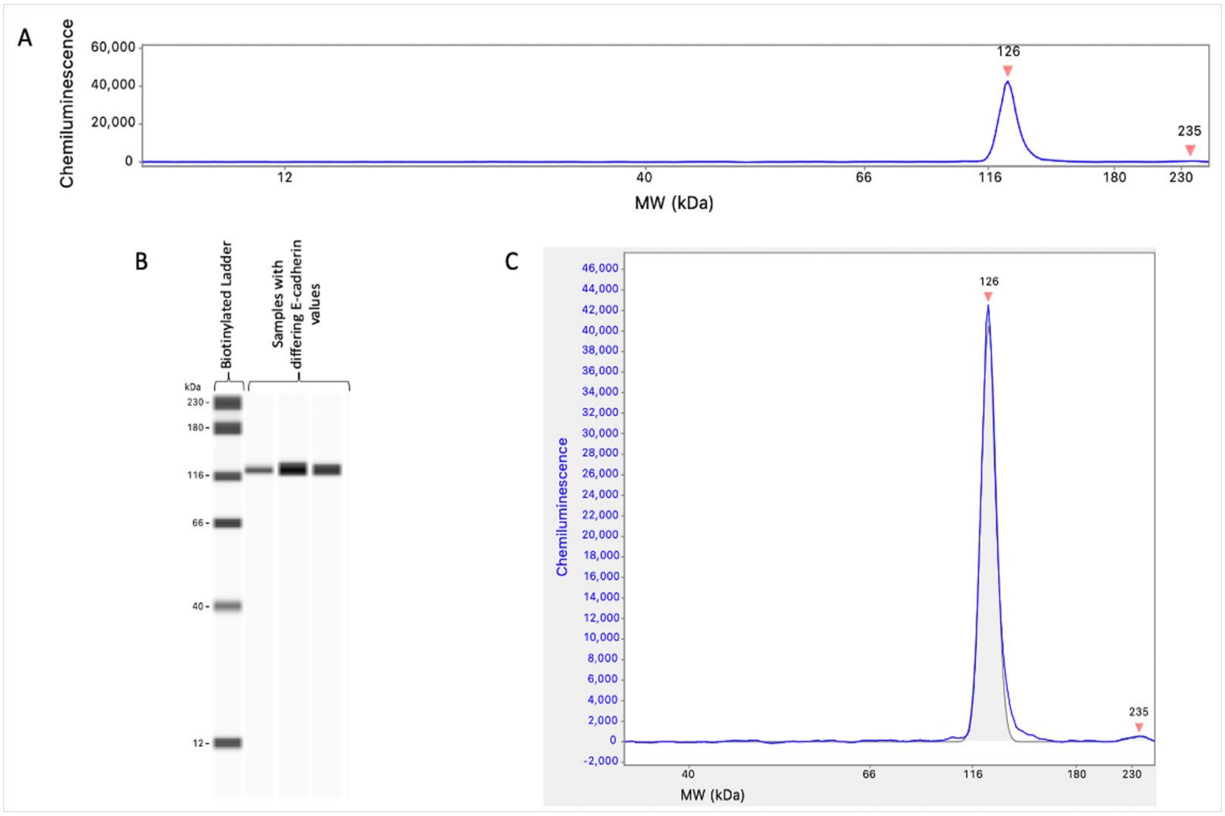


**Figure S1:** Visual demonstration of the output and interpretation of the Simple Western WES machine results, interpreted using Compass Software Version 5.0.1. (A) electropherogram produced by WES machine demonstrating single band for E-cadherin. (B) Virtual results from WES demonstrated in classical Western Blot format, as rendered by Protein Simple's Compass Software. (C) Image demonstrating electropherogram, with light grey shading outlining the fitting of peaks, used to estimate area under the curve and protein quantification.

**Table S3.** Reagents used for immunostaining of paraffin-embedded islet sections

| Antibody | Dilution | Source |
|---|---|---|
| Guinea pig anti-pig insulin | 1:1000 | Cedarlane Laboratories, Burlington, ON, Canada |
| Mouse anti-human E-cadherin antibody (4AC7) | 1:25 | ThermoFisher Scientific, Rockford, IL, USA |
| Mouse anti-human SNAP25 (ab11102) | 1:200 | Abcam, Cambridge, UK |
| Mouse anti-chicken alpha-tubulin antibody (ab7291) | 1:200 | Abcam |
| Biotinylated goat anti-mouse IgG (115065166) | 1:5000 | Jackson ImmunoResearch Laboratories, West Grove, PA, USA |
| Goat anti-guinea pig IgG (H+L) highly cross-adsorbed secondary antibody, Alexa Fluor 568 (A11075) | 1:200 | ThermoFisher Scientific |
| Goat anti-Mouse IgG (H+L) highly cross-adsorbed secondary antibody, Alexa Fluor 488 (A11029) | 1:200 | ThermoFisher Scientific |

**Table S4.** KRBH Solution Recipe

| Reagent | mM | Added for 1000mL KRBH |
|---|---|---|
| NaCl | 115 | 6.720g |
| KCl | 5 | 0.372g |
| $NaHCO_3$ | 24 | 2.016g |
| $CaCl_2$. $2H_2O$ | 2.5 | 0.368g |
| $MgCl_2$. $6H_2O$ | 1 | 0.203g |
| HEPES | 10 | 2.383g |
| BSA | 0.5% | 5.0g |
| Deionized $H_2O$ | | 1000mL |
| PH to 7.32 at 37$^o$C<br>Glucose and KCl were added as required for conditions of interest | | |

**Table S5.** Average RNA Integrity Number (RIN) values of RNA extracted from islets across various days of culture

| Day of Culture | n | Average RIN ± SD |
|---|---|---|
| Day 0 | 8 | 2.2 ± 1.0 |
| Day 1 | 7 | 3.2 ± 2.9 |
| Day 3 | 12 | 6.2 ± 2.8 |
| Day 5 | 12 | 7.5 ± 2.3 |
| Day 7 | 14 | 7.7 ± 0.7 |

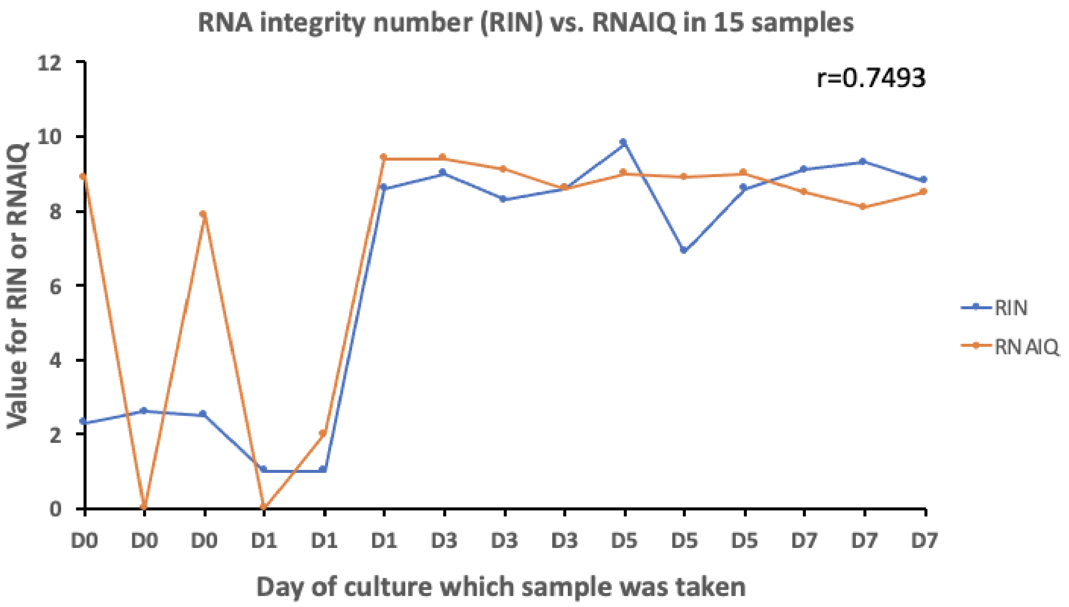


**Figure S2.** Comparison of two independent assessments used to determine RNA quality across 15 samples. RNA Integrity Number (RIN) values, as determined by Agilent 2100 Bioanalyzer (Agilent) vs. RNAIQ values, as determined by Qubit fluorometer (ThermoFisher Scientific).

**Table S6.** *CDH1* gene expression ratios in islets obtained from 1-, 3- and 7-day-old pigs

| **Pig ID** | **Days of Culture** | | | **P values** |
|---|---|---|---|---|
| | **Day 3 (control)** | **Day 5** | **Day 7** | |
| **1-day-old pig** | | | | 0.050 between groups (Kruskal Wallis) |
| 1 | 0.54 | 0.57 | 1.95 | |
| 2 | 0.76 | 2.12 | 2.04 | |
| 3 | 1.80 | 1.10 | 2.39 | |
| 4 | 1.36 | 1.66 | 2.29 | |
| Mean | 1.12 | 1.36 | 2.17 | |
| SD | 0.57 | 0.67 | 0.21 | |
| **3-day-old pig** | | | | 0.150 between groups (Kruskal Wallis) |
| 1 | 1.24 | 0.71 | 0.84 | |
| 2 | 0.87 | 0.43 | 0.84 | |
| 3 | 0.93 | 0.53 | 0.55 | |
| 4 | 0.32 | 0.73 | 0.74 | |
| 5 | 0.13 | 0.54 | 0.99 | |
| 6 | 0.69 | 0.72 | 1.02 | |
| Mean | 1.24 | 0.71 | 0.84 | |
| SD | 0.87 | 0.43 | 0.84 | |
| **7-day-old pig** | | | | 0.015 between groups (Kruskal Wallis)<br>0.032 D5 vs D7 |
| 1 | 1.20 | 1.53 | 1.46 | |
| 2 | 1.26 | 0.90 | 1.53 | |
| 3 | 1.12 | 1.20 | 1.66 | |
| 4 | 1.21 | 0.72 | 1.73 | |
| Mean | 1.20 | 1.09 | 1.60 | |
| SD | 0.06 | 0.36 | 0.12 | |

**Table S7.** CDH1 protein expression (chemiluminescence values) in islets obtained from 1-, 3- and 7-day-old pigs

| **Pig ID** | **Days of Culture** | | | **P values** |
|---|---|---|---|---|
| | **Day 3 (control)** | **Day 5** | **Day 7** | |
| **1-day-old pig** | | | | 0.019 between groups (Kruskal Wallis)<br>0.027 D3 vs D5 |
| 1 | 136503.90 | 365481.90 | 362444.20 | |
| 2 | 119915.00 | 150156.80 | 223167.90 | |
| 3 | 102947.70 | 194835.50 | 142385.50 | |
| 4 | 58471.00 | 147895.90 | 101788.30 | |
| 5 | 112179.00 | 242617.30 | 208983.30 | |
| 6 | 106738.00 | 179774.60 | 220972.40 | |
| 7 | 220637.70 | 248225.80 | 236929.50 | |
| 8 | 202634.60 | 346274.40 | 352957.20 | |
| Mean | 132503.4 | 234407.8 | 231203.5 | |
| SD | 53840.8 | 83739.5 | 90538.7 | |
| **3-day-old pig** | | | | 0.229 between groups (Kruskal Wallis) |
| 1 | 88023.1 | 349503. | 324552. | |
| 2 | 0. | 255322. | 394071.5 | |
| 3 | 403162.5 | 237000.6 | 212746.4 | |
| 4 | 206821.5 | 608347.7 | 581183.3 | |
| 5 | 719355.5 | 478622.8 | 332536.5 | |
| 6 | 103696.1 | 192340.3 | 299571.4 | |
| 7 | 253754.1 | 358436.8 | 359053.1 | |
| 8 | 290203.6 | 313969.9 | 361143.6 | |
| Mean | 258127.1 | 349192.9 | 358107.2 | |
| SD | 225787.9 | 136926.0 | 105154.7 | |
| **7-day-old pig** | | | | 0.698 between groups (Kruskal Wallis) |
| 1 | 144742.9 | 289979. | 143323.4 | |
| 2 | 52857.6 | 168310.3 | 150242.3 | |
| 3 | 79581.5 | 86407.2 | 128718.1 | |
| 4 | 67350.6 | 39947.3 | 186087.1 | |
| 5 | 328848.1 | 334916.4 | 290542.5 | |
| 6 | 244430.9 | 274917.2 | 172885.3 | |
| 7 | 87400.6 | 69800.9 | 87344.1 | |
| 8 | 138676.3 | 186289.8 | 23053.3 | |
| Mean | 142986.1 | 181321.0 | 147774.5 | |
| SD | 96961.8 | 110701.8 | 77531.0 | |

**Table S7 Continued**. Simulated western blot gels created from Protein Simple Wes Machine demonstrating E-cadherin protein expression (chemiluminescence values) outlined in Table S7 in islets obtained from 1-, 3- and 7-day-old pigs

**A** 1-day-old pigs, multiple samples demonstrating CDH1 expression at day 3 (column 4, 9, 14, 19), day 5 (column 5, 10, 15, 20) and day 7 (column 6, 11, 16, 21) of culture

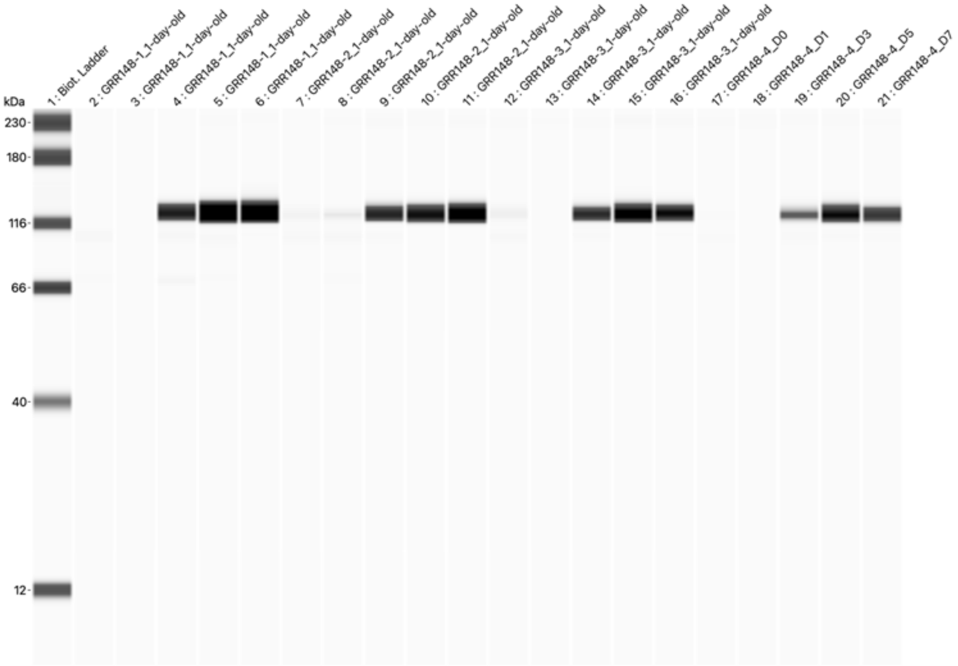


**B** 1-day-old pigs, multiple samples demonstrating E-cadherin expression at day 3 (column 4, 9, 14, 19), day 5 (column 5, 10, 15, 20) and day 7 (column 6, 11, 16, 21) of culture

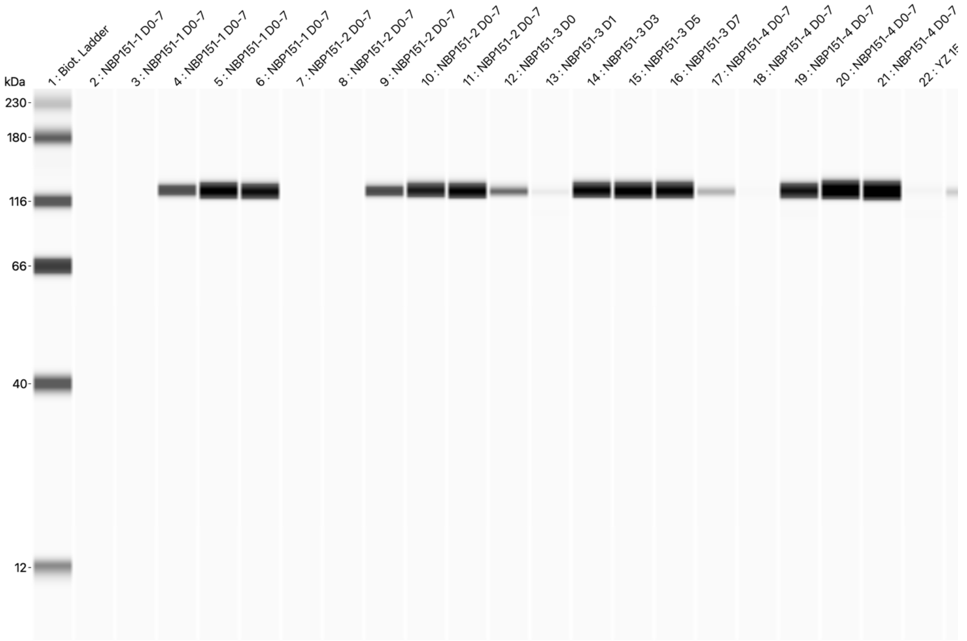

**C** 3-day-old pigs, multiple samples demonstrating E-cadherin expression at day 3 (columns 4, 9, 14, 19), day 5 (columns 5, 10, 15, 20) and day 7 (columns 6, 11, 16, 21) of culture

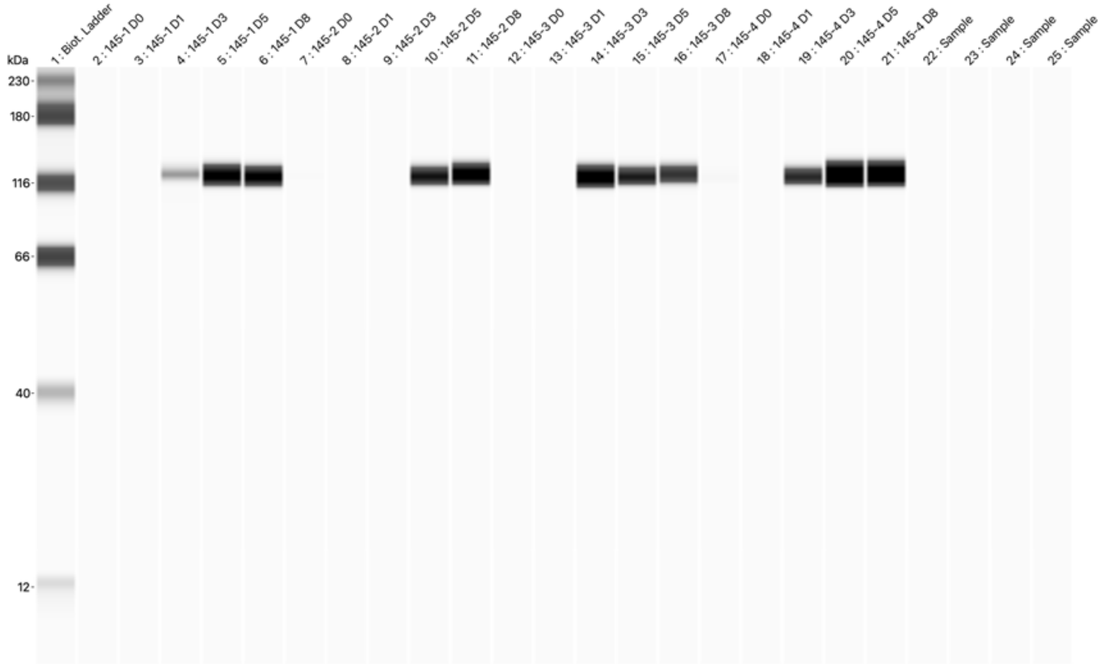


**D** 3-day-old pigs, multiple samples demonstrating E-cadherin expression at day 3 (columns 4, 9, 14, 19), day 5 (columns 5, 10, 15, 20) and day 7 (columns 6, 11, 16, 21) of culture

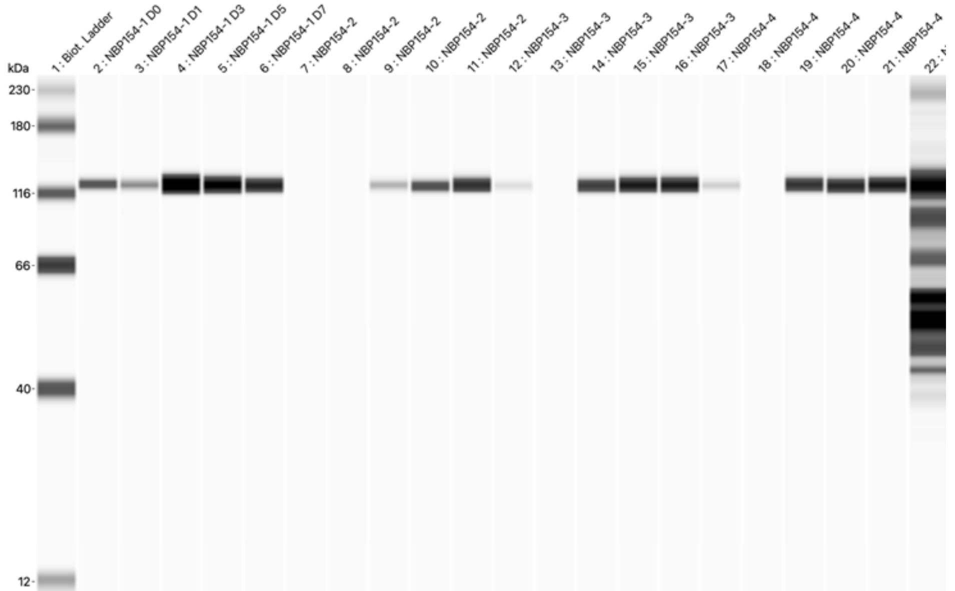


**E** 7-day-old pigs, multiple samples demonstrating E-cadherin expressions at day 3 (columns 4, 9, 14, 19), day 5 (columns 5, 10, 15, 20) and day 7 (columns 6, 11, 16, 21) of culture

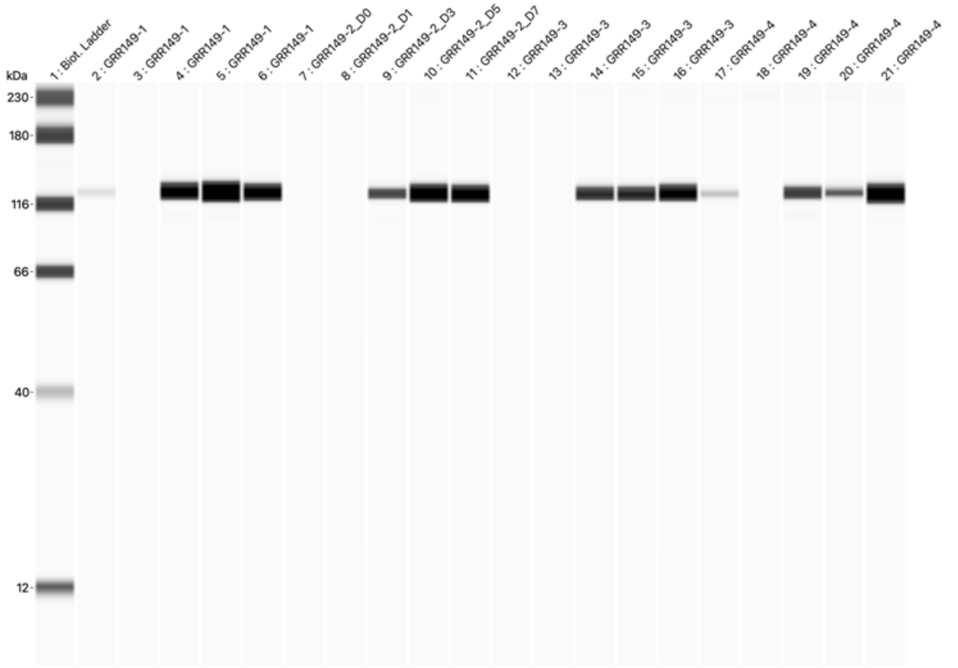


**F** 7-day-old pigs, multiple samples demonstrating E-cadherin expression at day 3 (columns 4,9,14,19), day 5 (columns 5,10,15,20) and day 7 (columns 6,11,16,21) of culture

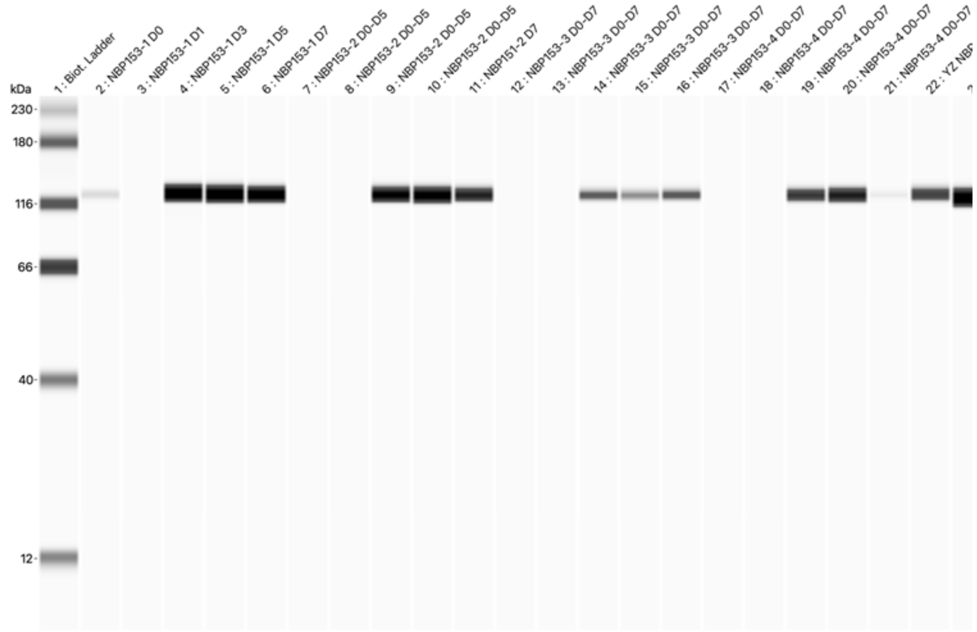

**Table S8.** *RAC1* gene expression ratios in islets obtained from 1-, 3- and 7-day-old pigs

| **Pig ID** | **Days of Culture** | | | **P values** |
|---|---|---|---|---|
| | **Day 3 (control)** | **Day 5** | **Day 7** | |
| **1-day-old pig** | | | | 0.443 between groups (Kruskal Wallis) |
| 1 | 0.93 | 1.08 | 0.49 | |
| 2 | 0.82 | 0.94 | 0.89 | |
| 3 | 1.21 | 1.34 | 1.00 | |
| 4 | 1.08 | 1.30 | 1.42 | |
| Mean | 1.01 | 1.17 | 0.95 | |
| SD | 0.17 | 0.19 | 0.38 | |
| **3-day-old pig** | | | | 0.332 between groups (Kruskal Wallis) |
| 1 | 1.09 | 1.01 | 0.90 | |
| 2 | 1.20 | 1.05 | 1.17 | |
| 3 | 1.11 | 1.29 | 1.35 | |
| 4 | 0.64 | 0.96 | 1.05 | |
| 5 | 0.98 | 0.99 | 1.28 | |
| 6 | 1.11 | 1.00 | 1.27 | |
| Mean | 1.09 | 1.01 | 0.90 | |
| SD | 1.20 | 1.05 | 1.17 | |
| **7-day-old pig** | | | | 0.746 between groups (Kruskal Wallis) |
| 1 | 0.48 | 0.38 | 0.32 | |
| 2 | 1.35 | 1.23 | 1.09 | |
| 3 | 1.44 | 1.60 | 1.34 | |
| 4 | 1.23 | 1.22 | 1.71 | |
| 5 | 0.88 | 1.07 | 2.08 | |
| Mean | 1.08 | 1.10 | 1.31 | |
| SD | 0.48 | 0.38 | 0.32 | |

**Table S9.** *Insulin* gene expression ratios in islets obtained from 1-, 3-, and 7-day-old pigs

| Pig ID | Days of Culture | | | P values |
|---|---|---|---|---|
| | Day 3 (control) | Day 5 | Day 7 | |
| **1-day-old pig** | | | | 0.122 between groups (Kruskal Wallis) |
| 1 | 0.89 | 2.13 | 2.76 | |
| 2 | 0.63 | 1.20 | 1.27 | |
| 3 | 1.02 | 1.62 | 1.78 | |
| 4 | 1.75 | 1.99 | 1.65 | |
| Mean | 1.07 | 1.74 | 1.87 | |
| SD | 0.48 | 0.42 | 0.63 | |
| **3-day-old pig** | | | | 0.121 between groups (Kruskal Wallis) |
| 1 | 0.64 | 3.42 | 1.28 | |
| 2 | 0.71 | 1.5 | 1.49 | |
| 3 | 1.02 | 5.28 | 3.55 | |
| 4 | 3.26 | 1.17 | 0.36 | |
| 5 | 1.27 | 2.97 | 0.91 | |
| 6 | 0.52 | 1.10 | 0.85 | |
| Mean | 0.64 | 3.42 | 1.28 | |
| SD | 0.71 | 1.5 | 1.49 | |
| **7-day-old pig** | | | | 0.763 between groups (Kruskal Wallis) |
| 1 | 0.30 | 0.27 | 0.26 | |
| 2 | 1.26 | 1.97 | 1.78 | |
| 3 | 2.01 | 2.39 | 2.64 | |
| 4 | 1.02 | 0.92 | 1.78 | |
| 5 | 1.31 | 0.48 | 1.63 | |
| Mean | 1.18 | 1.21 | 1.62 | |
| SD | 0.30 | 0.27 | 0.26 | |

**Table S10.** *SNAP25* gene expression ratios in islets obtained from 1-, 3-, and 7-day-old pigs

| Pig ID | Days of Culture | | | P values |
|---|---|---|---|---|
| | Day 3 (control) | Day 5 | Day 7 | |
| **1-day-old pig** | | | | 0.026 between groups (Kruskal Wallis)<br>0.028 D5 vs D7 |
| 1 | 0.64 | 0.14 | 1.42 | |
| 2 | 0.53 | 1.17 | 2.32 | |
| 3 | 1.34 | 0.30 | 3.79 | |
| 4 | 2.24 | 0.71 | 2.63 | |
| Mean | 1.18 | 0.58 | 2.54 | |
| SD | 0.79 | 0.46 | 0.98 | |
| **3-day-old pig** | | | | 0.004 between groups (Kruskal Wallis)<br>0.002 D5 vs D7 |
| 1 | 1.65 | 2.82 | 3.39 | |
| 2 | 1.41 | 1.70 | 3.04 | |
| 3 | 2.21 | 2.53 | 5.02 | |
| 4 | 0.54 | 1.92 | 3.16 | |
| 5 | 0.39 | 1.76 | 2.39 | |
| 6 | 0.92 | 1.66 | 2.16 | |
| Mean | 1.19 | 2.07 | 3.19 | |
| SD | 0.70 | 0.49 | 1.01 | |
| **7-day-old pig** | | | | 0.309 between groups (Kruskal Wallis) |
| 1 | 1.62 | 2.86 | 4.01 | |
| 2 | 1.62 | 2.18 | 3.31 | |
| 3 | 3.78 | 4.84 | 14.11 | |
| 4 | 0.74 | 0.27 | 1.67 | |
| 5 | 0.14 | 0.09 | 1.54 | |
| Mean | 1.58 | 2.05 | 4.93 | |
| SD | 1.38 | 1.97 | 5.24 | |

**Table S11.** SNAP25 protein expression (chemiluminescence values) in islets obtained from 1-, 3-, and 7-day-old pigs at various time points in culture

| Pig ID | Days of Culture | | | P values |
|---|---|---|---|---|
| | Day 3 (control) | Day 5 | Day 7 | |
| **1-day-old pig** | | | | 0.017 between groups (Kruskal Wallis)<br>0.029 D3 vs D7 |
| 1 | 144361.9 | 70883.0 | 0.00 | |
| 2 | 228135.8 | 102468.3 | 52233.8 | |
| 3 | 76177.2 | 56826.9 | 52294.9 | |
| 4 | 160430.7 | 118251.7 | 67615.2 | |
| Mean | 152276.4 | 87107.5 | 43036.0 | |
| SD | 62382.9 | 28202.1 | 29589.2 | |
| **3-day-old pig** | | | | 0.309 between groups (Kruskal Wallis) |
| 1 | 83625.3 | 56361.2 | 96952.9 | |
| 2 | 89143.9 | 111335.3 | 42855.9 | |
| 3 | 351403.2 | 192495.1 | 54747.8 | |
| 4 | 166190.4 | 202361.5 | 155978.9 | |
| Mean | 172590.7 | 140638.3 | 87633.9 | |
| SD | 125024.1 | 69426.7 | 51135.3 | |
| **7-day-old pig** | | | | 0.219 between groups (Kruskal Wallis) |
| 1 | 91607.9 | 207298.8 | 59190.8 | |
| 2 | 53652.7 | 112513.9 | 93947.9 | |
| 3 | 873036.8 | 108924.6 | 64065.7 | |
| 4 | 173930.9 | 85786.9 | 75415.9 | |
| Mean | 298057.1 | 128631.1 | 73155.1 | |
| SD | 386593.6 | 53766.0 | 15438.8 | |

**Table S11 Continued**. Simulated western blot gels created from Protein Simple Wes Machine demonstrating SNAP25 protein expression (chemiluminescence values) outlined in Table S11 in islets obtained from 1-, 3-, and 7-day-old pigs

**A** 1-day-old pigs, multiple samples demonstrate SNAP25 expression at day 3 (columns 6, 7, 16, 17), day 5 (columns 8, 9, 18, 19) and day 7 (columns 10, 11, 20, 21) of culture

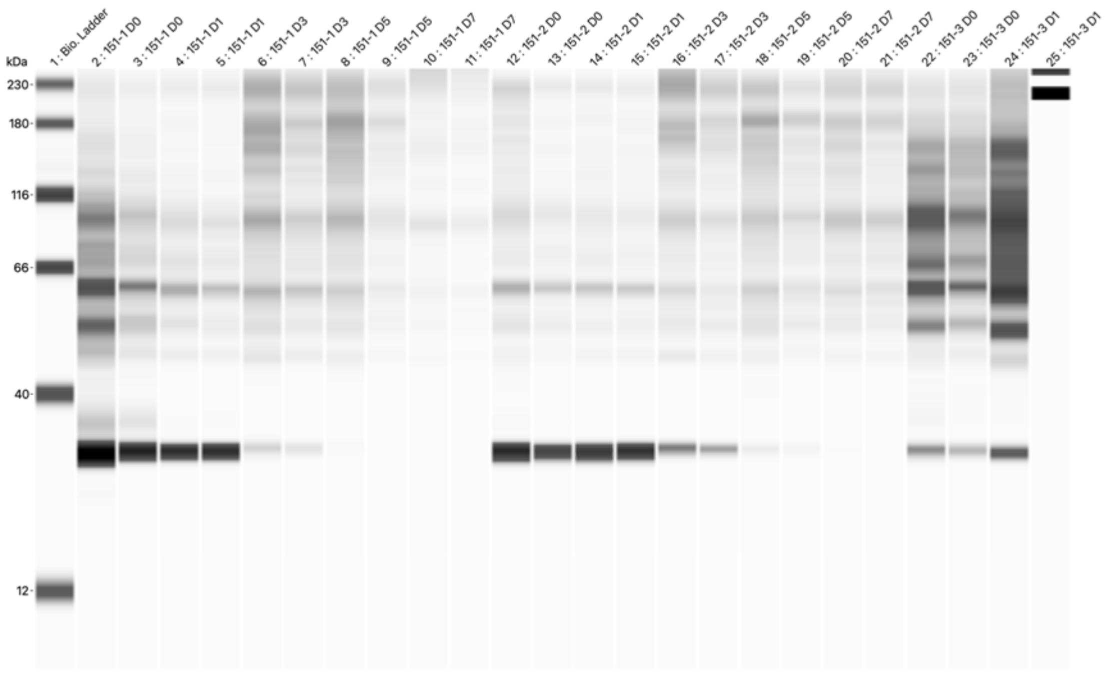


**B** 1-day-old pigs, multiple samples demonstrating SNAP25 expressions on day 3 (columns 2, 3, 12, 13), day 5 (columns 4, 5, 14, 15) and day 7 (columns 6, 7, 16, 17) of culture and 3-day-old pigs, multiple samples demonstrating SNAP 25 expression at day 3 (columns 22, 23) and day 5 (24, 25) of culture

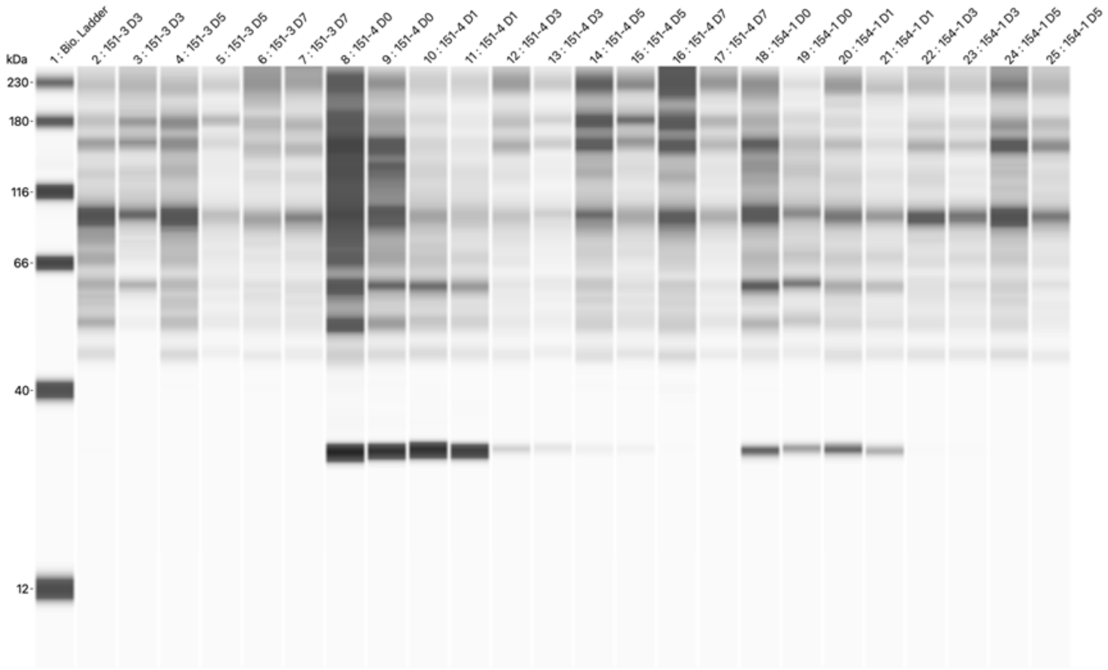

**C** 3-day-old pigs, multiple samples demonstrating SNAP25 expressions at day 3 (columns 6, 7, 16, 17), day 5 (columns 8, 9, 18, 19) and day 7 (columns 10, 11, 20, 21) of culture

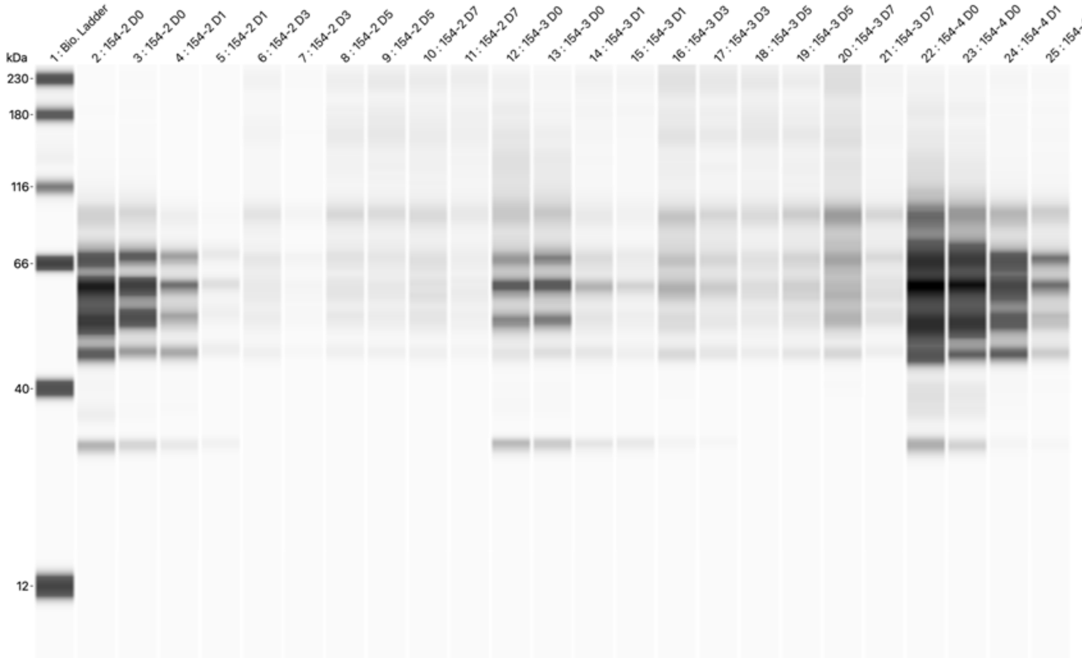


**D** 3-day-old pigs, multiple samples demonstrating SNAP25 expression on day 3 (columns 24, 25), day 7 (columns 22, 23) and 7-day-old pigs, multiple samples demonstrating SNAP 25 expression at day 3 (columns 6, 7, 16, 17), day 5 (columns 8, 9, 18, 19) and day 7 (columns 10, 11, 20, 21) of culture

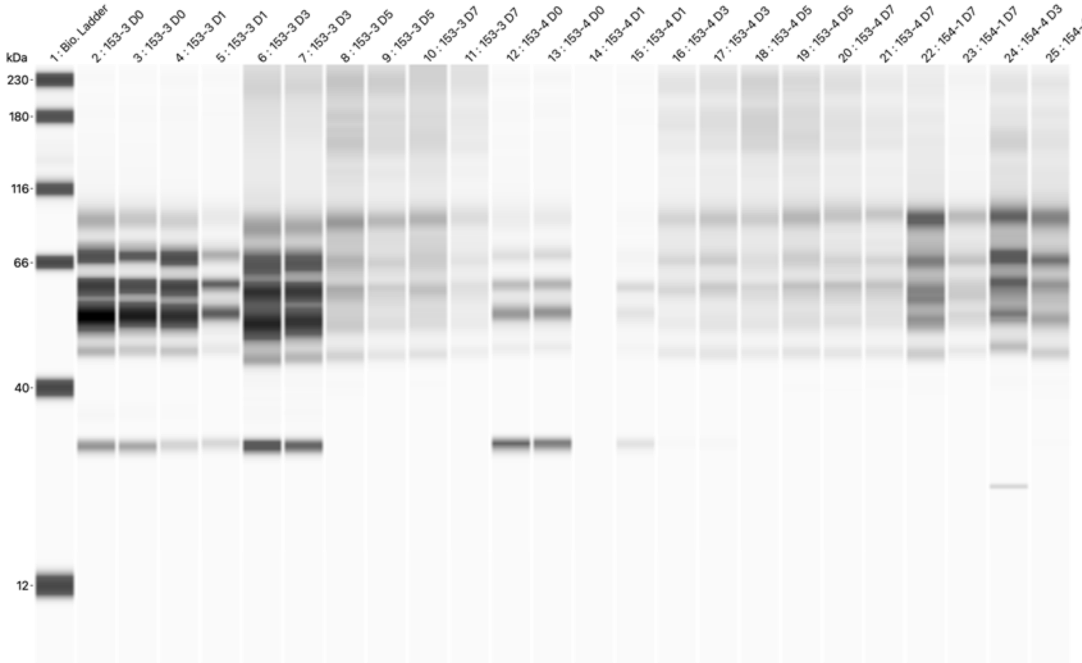


**E** 3-day-old pigs, multiple samples demonstrating SNAP25 expression on day 5 (columns 22, 23), day 7 (columns 24, 25) and 7-day-old pigs, multiple samples demonstrating SNAP 25 expression at day 3 (columns 6, 7, 16, 17), day 5 (columns 8, 9, 18, 19) and day 7 (columns 10, 11, 20, 21) of culture

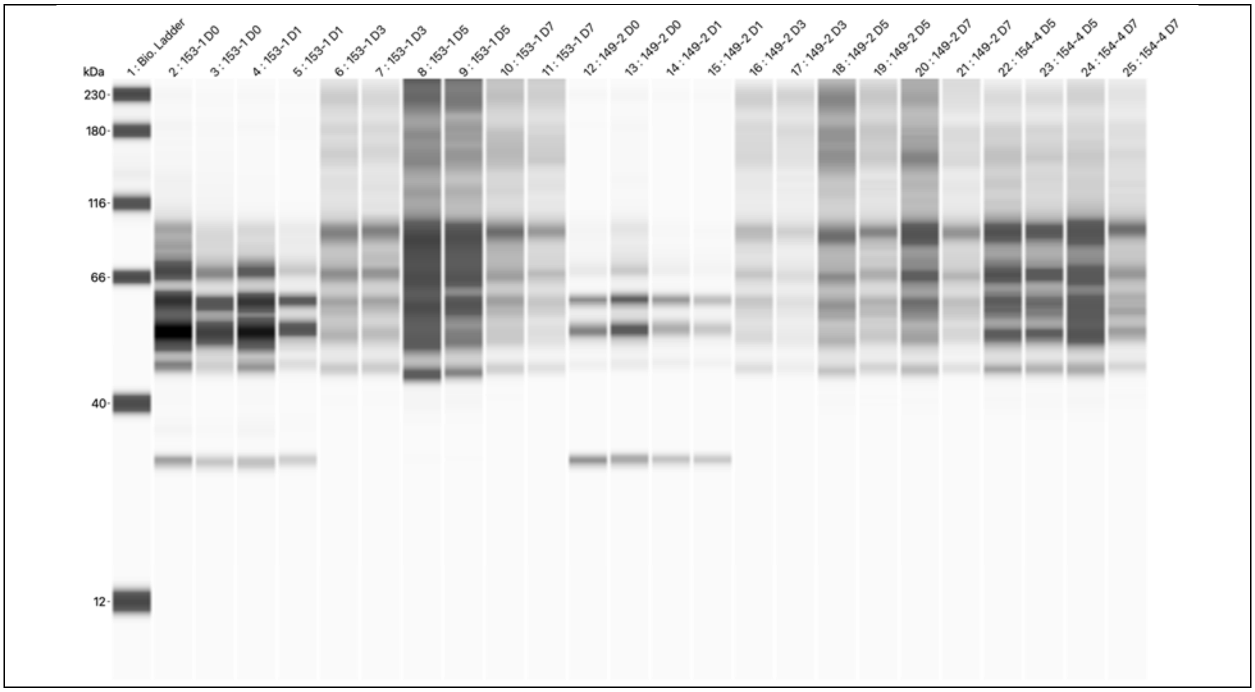
kDa
1 : Bio. Ladder
2 : 153-1 D0
3 : 153-1 D0
4 : 153-1 D1
5 : 153-1 D1
6 : 153-1 D3
7 : 153-1 D3
8 : 153-1 D5
9 : 153-1 D5
10 : 153-1 D7
11 : 153-1 D7
12 : 149-2 D0
13 : 149-2 D0
14 : 149-2 D1
15 : 149-2 D1
16 : 149-2 D3
17 : 149-2 D3
18 : 149-2 D5
19 : 149-2 D5
20 : 149-2 D7
21 : 149-2 D7
22 : 154-4 D5
23 : 154-4 D5
24 : 154-4 D7
25 : 154-4 D7
230
180
116
66
40
12

**Table S12.** Amount of insulin secreted (over total insulin content) expressed as percent insulin secreted by islets obtained from 1-, 3-, and 7-day-old pigs following *in vitro* glucose stimulation assay

| Pig ID | KRBH with 2.8mM Glucose | KRBH with 20mM Glucose | KRBH with 20mM Glucose plus 30mM KCl | P values |
|---|---|---|---|---|
| **1-day-old pig** | | | | 0.037 between groups (Kruskal Wallis) |
| 1 | 0.22 | 1.14 | 0.77 | |
| 2 | 0.54 | 3.58 | 4.23 | |
| 3 | 0.88 | 3.31 | 3.10 | |
| 4 | 0.58 | 1.76 | 1.62 | |
| Mean | 0.56 | 2.45 | 2.43 | |
| SD | 0.26 | 1.18 | 1.53 | |
| **3-day-old pig** | | | | 0.0273 between groups (Kruskal Wallis)<br>0.380 between 2.8mM glucose and 20mM glucose plus 30mM KCl |
| 1 | 0.08 | 0.55 | 3.83 | |
| 2 | 0.42 | 1.35 | 1.89 | |
| 3 | 0.19 | 0.67 | 1.75 | |
| Mean | 0.23 | 0.86 | 2.49 | |
| SD | 0.17 | 0.43 | 1.16 | |
| **7-day-old pig** | | | | 0.038 between groups (Kruskal Wallis)<br>0.450 between 2.8mM glucose and 20mM glucose plus 30mM KCl |
| 1 | 0.23 | 1.39 | 2.25 | |
| 2 | 0.36 | 1.23 | 1.65 | |
| 3 | 0.17 | 0.99 | 3.24 | |
| 4 | 1.65 | 2.60 | 3.88 | |
| Mean | 0.60 | 1.55 | 2.44 | |
| SD | 0.70 | 0.35 | 0.66 | |

**Table S13.** Amount of insulin secreted (over total insulin content) expressed as percent insulin secreted by untreated or E-cadherin antibody-treated islets obtained from 3-day-old pigs following *in vitro* glucose stimulation assay

| Conditions and Pig ID | KRBH with 2.8mM Glucose | KRBH with 20mM Glucose | Stimulation Index | P values |
|---|---|---|---|---|
| **Untreated** | | | 2.8 | 0.250 between groups (Wilcoxon matched pairs signed rank test) |
| 1 | 0.29 | 0.54 | | |
| 2 | 0.39 | 1.54 | | |
| 3 | 0.22 | 0.44 | | |
| Mean | 0.30 | 0.84 | | |
| SD | 0.08 | 0.61 | | |
| **Treated** | | | 1.2 | 0.462 between groups (Wilcoxon matched pairs signed rank test) |
| 1 | 0.33 | 0.32 | | |
| 2 | 0.34 | 0.49 | | |
| 3 | 0.15 | 0.15 | | |
| Mean | 0.27 | 0.32 | | |
| SD | 0.10 | 0.17 | | |
